# Tunable Trimers: Using Temperature and Pressure to Control Luminescent Emission in Gold(I) Pyrazolate-Based Trimers

**DOI:** 10.1002/chem.201404058

**Published:** 2014-10-21

**Authors:** Christopher H Woodall, Sara Fuertes, Christine M Beavers, Lauren E Hatcher, Andrew Parlett, Helena J Shepherd, Jeppe Christensen, Simon J Teat, Mourad Intissar, Alexandre Rodrigue-Witchel, Yan Suffren, Christian Reber, Christopher H Hendon, Davide Tiana, Aron Walsh, Paul R Raithby

**Affiliations:** [a] Department of Chemistry, University of BathBath BA2 7AY (UK); [b] Station 11.3.1 Advanced Light Source, Lawrence Berkeley National LaboratoryBerkeley, CA 94720 (USA); [c] Departement de Chimie, Université de MontréalMontréal, QC, H3C 3J7 (Canada)

**Keywords:** aurophilicity, crystallography, gold, high pressure, luminescence

## Abstract

A systematic investigation into the relationship between the solid-state luminescence and the intermolecular Au⋅⋅⋅Au interactions in a series of pyrazolate-based gold(I) trimers; tris(μ_2_-pyrazolato-*N*,*N*′)-tri-gold(I) (**1**), tris(μ_2_-3,4,5- trimethylpyrazolato-*N*,*N*′)-tri-gold(I) (**2**), tris(μ_2_-3-methyl-5-phenylpyrazolato-*N*,*N*′)-tri-gold(I) (**3**) and tris(μ_2_-3,5-diphenylpyrazolato-*N*,*N*′)-tri-gold(I) (**4**) has been carried out using variable temperature and high pressure X-ray crystallography, solid-state emission spectroscopy, Raman spectroscopy and computational techniques. Single-crystal X-ray studies show that there is a significant reduction in the intertrimer Au⋅⋅⋅Au distances both with decreasing temperature and increasing pressure. In the four complexes, the reduction in temperature from 293 to 100 K is accompanied by a reduction in the shortest intermolecular Au⋅⋅⋅Au contacts of between 0.04 and 0.08 Å. The solid-state luminescent emission spectra of **1** and **2** display a red shift with decreasing temperature or increasing pressure. Compound **3** does not emit under ambient conditions but displays increasingly red-shifted luminescence upon cooling or compression. Compound **4** remains emissionless, consistent with the absence of intermolecular Au⋅⋅⋅Au interactions. The largest pressure induced shift in emission is observed in **2** with a red shift of approximately 630 cm^−1^ per GPa between ambient and 3.80 GPa. The shifts in all the complexes can be correlated with changes in Au⋅⋅⋅Au distance observed by diffraction.

## Introduction

Homoleptic gold(I) trimers are an intriguing class of photoemissive materials. They frequently possess bright phosphorescence and “hexamer” style molecular geometries that are commonly associated with the presence of Au⋅⋅⋅Au “aurophilic interactions” that dominate the solid-state chemistry of gold(I).^1^ As a group of compounds they demonstrate many desirable properties such as a tuneable emission wavelength and a sensitivity to environmental conditions that makes them promising candidates for application as imaging agents or sensors.1a, b, 2 Aurophilic interactions are the result of relativistic effects and impart solid-state stabilisation energies in the range 7–12 kcal mol^−1^, comparable with those attributed to hydrogen bonds.1d Aurophilic interactions are generally considered to be less directional than conventional hydrogen bonds. Extensive synthetic, structural, theoretical and photophysical studies have been performed on a variety of gold(I) trimers including pyrazolates, pyridinates, imidazolates, carbeniates and triazolates, all of which consistently demonstrate the importance of aurophilic interactions in the solid-state for the observations of their photoluminescent properties.^2^, ^3^

For example, the examination of three polymorphs of Au_3_(MeN=COMe)_3_ by Balch et al. clearly demonstrates the effects of altering aurophilic interactions in gold(I) trimers with each form displaying a distinct emission depending on the aurophilic interactions present.3d The hexagonal phase of Au_3_(MeN=COMe)_3_ displays a unique solvo-luminescence (*λ*_ex_=366 nm, *λ*_em_=520 nm) that has been attributed to its columnar crystal structure and the ease with which solvent molecules may perturb aurophilic interactions within. The other two forms display more typical luminescent properties (triclinic phase *λ*_em_=444 nm; monoclinic phase *λ*_em_=420 nm).3b, c Emissive behaviour is absent in solution studies of the compound and in structural analogues where intermolecular aurophilic interactions have been disrupted beyond the length of 3.6 Å, through steric interactions. Theoretical work has confirmed their role in the metal-to-metal (MMCT) and metal-to-ligand (MLCT) charge transfer transitions involved in the luminescent emissions.3b, d, m, 4

Intermolecular aurophilic interactions have been shown to be highly sensitive to conditions such as temperature with several examples of gold(I) trimers displaying thermochromism.^2^, ^5^ The triazolate-based trimer investigated by Coppens and Omary et al.^2^ displays a shift in emission wavelength upon cooling from approximately 725 nm at 280 K (*λ*_ex_=280 nm) to 755 nm at 90 K. The shift in emission can be attributed to a reduction in intermolecular aurophilic interaction length from 3.42 Å at room temperature to 3.19 Å at 90 K. Time resolved photocrystallographic studies of the copper analogue have observed similar structural distortions in the excited state, confirming the temperature induced structural distortion is directly linked to the change in emission spectrum.^6^ Other examples include [μ_3_-S(AuCNC_7_H_13_)_3_](SbF_6_), a sulfur-capped gold(I) trimer complex that aggregates into a hexamer with a staggered geometry in the solid state. The complex undergoes a reversible phase transition at 150 K between a low temperature monoclinic phase and a high temperature orthorhombic phase. At the transition to the monoclinic phase the hexamer resolves into two crystallographically independent sites as opposed to the one present at high temperature. The two hexamers have dramatically different intermolecular aurophilic interaction lengths of approximately 3.72 Å and about 3.38 Å, if averaged, opposed to the approximately 3.56 Å of the high-temperature phase. The change in aurophilic interaction lengths coincides with changes in the emission spectra, the single band emission of the high temperature phase (*λ*_em_=678 nm) resolving into two different peaks each associated with one of the two hexamers in the low temperature phase (490 and 680 nm).3e

The application of hydrostatic pressure is a well-established tool for altering the thermodynamics of a system and has been used to induce unique behaviour in a range of materials.^7^ Gold compounds have been attracting attention because of the unusual mechanochromic properties and negative linear compressibility that they display under high pressures.^8^ Despite this there has been very limited use of high pressure to investigate changes in aurophilic interactions and the correlation with changes in luminescence.^9^ In a rare example dicyanoaurates with a lamellar structure have been shown to red shift in the range of 1200–2000 cm^−1^ per GPa depending on the charge balancing cation.^10^ There are currently no structural examples of gold(I) trimers having been investigated at high pressure; however, work by Reber et al. has demonstrated that hydrostatic pressure may be used to tune the wavelength of a luminescent emission in gold(I) dithiocarbamate systems.^11^ Luminescence spectra of dithiocarbamate complexes of gold(I) show an emission band at 18 000 cm^−1^ at room temperature. The band energy is strongly affected by external pressure, with a red shift of the maxima of −1200 cm^−1^ per GPa observed. Computational work on clusters of the gold(I) thiocarbamate systems suggests that a compression of the length of the intermolecular aurophilic interactions results in reduction in the electronic band gap (BG) and the associated red shift in the emission.^11^

**Scheme 1:**
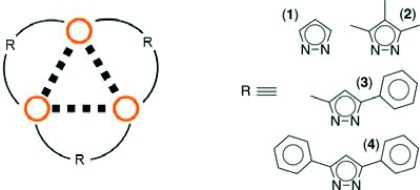
Molecular structure of the four gold(I) trimers investigated, tris(μ_2_-pyrazolato-*N*,*N*′)-tri-gold(I) (1), tris(μ_2_-3,4,5-trimethylpyrazolato-*N*,*N*′)-tri-gold(I) (2), tris(μ_2_-3-methyl-5-phenylpyrazolato-*N*,*N*′)-tri-gold(I) (3) and tris(μ_2_-3,5-diphenylpyrazolato-*N*,*N*′)-tri-gold(I) (4); ○=Au^I^ atom.

**Figure 1:**
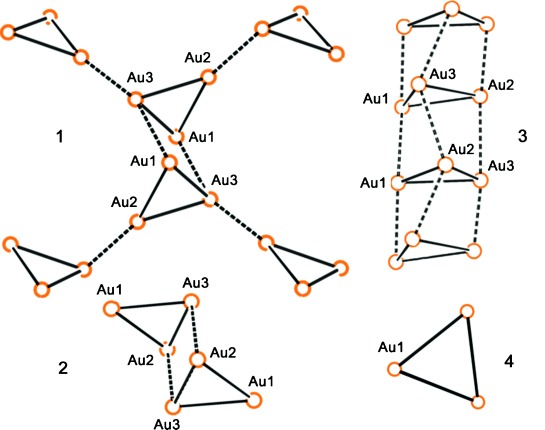
Aurophilic interactions present in compounds 1–4 (- intratrimer; — intertrimer; ○=Au^I^ atom). Compounds 1 and 2 display strong luminescence with intermolecular aurophilic interactions of approximately 3.3 Å. Compound 3 displays no luminescence at RT with long aurophilic interactions of about 3.6 Å. Compound 4 displays no luminescence.

From these studies it is apparent that variations in both temperature and pressure on polynuclear gold complexes can be used to control their luminescent properties. However, to date, the examples in the literature have been on isolated complexes, and there have been few comparisons of the effect of both temperature and pressure on the same chemical systems. We now report the first systematic solid-state study of temperature and pressure effects, and the correlation with luminescence, on a series of gold(I) trimeric complexes. We have chosen four gold(I) pyrazolate complexes and used a combination of complementary techniques, including variable temperature and high pressure single crystal X-ray crystallography, solid-state luminescence spectroscopy, Raman spectroscopy and periodic density functional theory (DFT) calculations, to probe the structure–property relationship. Four gold(I) pyrazolate complexes (Scheme 1) were selected from the literature for the study based upon the similarity of their core molecular structure while they display a range of different aurophilic interactions (Figure 1) and photophysical properties.3a, j, 12

Crystal structures of compounds **1**–**3** show close intermolecular aurophilic interactions to form hexamers of varying geometries in the solid state (Figure 1). Compounds **1** and **2** exhibit intermolecular aurophilic contacts of approximately 3.3 Å linking trimers to form hexamers. Compound **3** exhibits a columnar structure that results in extended aurophilic interactions above and below individual trimers of approximately 3.67 Å and, therefore, too long for the observation of many of the photoluminescent properties associated with such interactions. The solid state structure of **4** displays no intermolecular aurophilic interactions at all.1

## Results and Discussion

Firstly, the structures of **1**–**4** were investigated using single crystal X-ray diffraction over the temperature range 293–100 K and then from ambient pressure to 7.81 GPa, sample permitting, in order to develop a greater understanding of how both temperature and pressure may effect such systems. As expected, all four display a contraction in unit-cell volume with a reduction in temperature (Table 1). Compound **3** undergoes the greatest contraction in unit cell volume with a reduction from 3140.9(7) to 3016.2(4) Å^3^ contracting at a rate of 0.162 Å^3^ per molecule, per unit cell, per degree [ΔÅ^3^ *Z*^−1^ K^−1^] while **4** undergoes the second greatest contraction at 0.13 ΔÅ^3^ *Z*^−1^ K^−1^. The size of unit-cell volume contraction is dependent on the size of void space present within the crystal structure. Compound **3** possesses a large void, 13.6 % of unit cell-volume as measured with the default parameters in the Mercury software^13^ (probe radius 1.2 Å, grid spacing 0.7 Å) occupied by disordered solvent as previously reported but not discussed by Yang et al.3a

**Table 1:** Unit cell volume with temperature.

*T*	Unit cell volume [Å^3^]			
[K]	1	2	3	4
293	1265.9(3))	2168.29(10)	3140.9(7)	5751.7(2)
270	1261.8(3)	2159.34(10)	3123.5(5)	5721.70(8)
240	1256.3(3)	2148.28(9)	3102.4(5)	5709.20(12)
210	1250.7(2)	2137.98(9)	3082.9(6)	5685.25(12)
180	1245.3(3)	2127.96(9)	3062.1(4)	5664.75(12)
150	1241.1(3)	2117.68(9)	3045.8(4)	5647.87(8)
120	1236.6(3)	2107.38(8)	3028.1(4)	5627.70(12)
100	1234.1(4)	2101.28(8)	3016.2(4)	5597.84(8)

It is the solvent cavity which undergoes greater contraction than the other components of the structure, contracting to 12.4 % of the structure at 100 K (see the Supporting Information). There is no direct correlation between this reduction in void space and a reduction in intermolecular Au⋅⋅⋅Au interactions. Calculations with Mercury show that **1** and **2** possess no void space and display slower contraction rates of 0.041 and 0.087 ΔÅ^3^ *Z*^−1^ K^−1^, respectively. Compound **4** possesses a small void (2.3 %), not previously discussed in the literature, and undergoes a smaller contraction than **3**.

**Figure 2:**
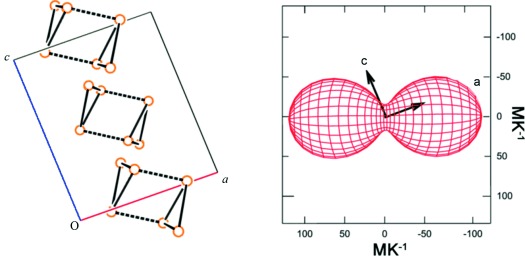
Left: packing of selected trimers in the crystal structure of 2 viewed down the *b* axis, all atoms except gold omitted for clarity. Dashed lines indicate intermolecular aurophilic interactions. Right: thermal indicatrix of 2 viewed down X3 axis of ellipsoid.

All four compounds display a natural propensity to contract in a manner that reduces the intertrimer stacking distances. This is demonstrated clearly for **2** through calculation of the principal axes of contraction relative to the crystallographic axes and relating the results to the unit-cell contents (Figure 2).

Similar behaviour is observed in the other three compounds (see the Supporting Information).^14^ The contraction coincides with the direction of intermolecular aurophilic interactions in **1**–**3** and reduces the distances between the gold centres by approximately 0.05 Å between 293–100 K (Table 2).

**Table 2:** Intermolecular aurophilic interactions with variable temperature for 1–3.

*T*	Intermolecular aurophilic interaction [Å]								
[K]		1			2			3	
	Au1–Au1′	Au1–Au2′	Au1–Au3′	Au1–Au2′	Au2–Au3′	Au3–Au1′	Au1–Au1′	Au2–Au3′	Au3–Au2′
293	3.7346(9)	4.4924(8)	3.3095(7)	3.3345(4)	4.7791(5)	5.1049(5)	3.7043(5)	3.6782(7)	3.8554(7)
270	3.7313(9)	4.4855(8)	3.3031(7)	3.3239(5)	4.7731(6)	5.0890(6)	3.6943(4)	3.6717(5)	3.8453(5)
240	3.7232(8)	4.4775(7)	3.2945(6)	3.3099(5)	4.7648(6)	5.0686(6)	3.6831(4)	3.6635(5)	3.8353(5)
210	3.7167(7)	4.4686(6)	3.2855(5)	3.2970(5)	4.7559(5)	5.0493(5)	3.6730(4)	3.6571(7)	3.8256(7)
180	3.7098(7)	4.4530(6)	3.2764(5)	3.2838(5)	4.7456(5)	5.0298(5)	3.6633(3)	3.6501(5)	3.8164(5)
150	3.7050(8)	4.4526(6)	3.2704(5)	3.2709(4)	4.7356(5)	5.0118(5)	3.6553(3)	3.6453(6)	3.8081(6)
120	3.6987(8)	4.4463(6)	3.2633(5)	3.2579(4)	4.7238(5)	4.9941(5)	3.6470(3)	3.6391(6)	3.8004(6)
100	3.695(1)	4.4421(8)	3.2587(7)	3.2494(4)	4.7172(5)	4.9836(4)	3.6422(3)	3.6368(5)	3.7944(5)

**Figure 3:**
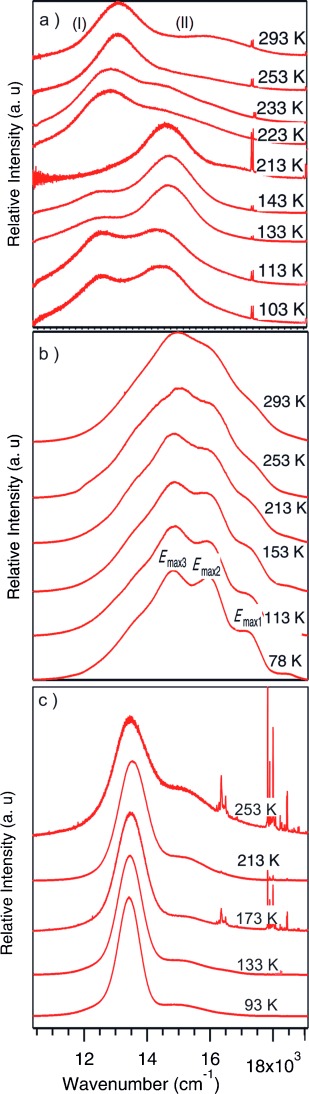
High-pressure emission spectra for: a) 1, b) 2, and c) 3.

Luminescence spectra were collected on crystalline samples of the four compounds over the temperature range 293–78 K. Compound **1** undergoes the most significant changes in the spectra (Figure 3 a). At room temperature the emission spectrum shows two broad bands (I and II) the most intense (I) centred at 13 160 cm^−1^ while the less intense peak appears at higher energy as a shoulder at 15 950 cm^−1^. Upon cooling, the presence of a new band at 14 670 cm^−1^ becomes apparent, first observed at 233 K and increasing in intensity, to become the most intense peak in the spectra from 213 K. At temperatures below 133 K the bands at 13 160 and 14 670 cm^−1^ have comparable intensities and a width at half height of approximately 2000 cm^−1^. Raman spectra collected under the same conditions show concomitant changes in the regions associated with Au–N and Au–Au interactions (see the Supporting Information).

Compound **2** displays a broad emission centred at approximately 15 000 cm^−1^, that, upon cooling, displays a more resolved structure with a minimal red shift for *E*_max_ (Figure 3 b). The band shape and vibronic spacing of about 1140 to 1200 cm^−1^ indicates an important ligand contribution to the transition while the minimal shift is similar to that reported for gold(I) diethyldithiocarbamate (1–2 cm^−1^ per K).^11^

As previously reported by Yang et al., **3** displays no luminescence at room temperature despite an average midrange Au–Au interaurophilic distance of 3.7460(11) Å. Upon cooling a featureless emission appears at 13 520 cm^−1^ becoming more symmetrical with decreasing temperature (Figure 3 c). The appearance of the emission band in **3** with decreasing temperature can be attributed to the reduction in intermolecular aurophilic distances which contract by 0.019 Å between 293 and 240 K (from 3.7460(11) to 3.7273(8) Å) and of 0.05 Å over the range studied. For **3** the *E*_max_ value versus temperature decreases linearly by approximately 1 cm^−1^ per K as temperature is reduced. A shoulder near 15 200 cm^−1^ is also present for which there is no significant change with temperature. No vibronic structure is observed at low temperature. The width at half height is about 1100 cm^−1^ at 253 K decreases with temperature to 820 cm^−1^ at 93 K.

Compound **4** displays no emission bands at any temperature confirming that presence of intramolecular aurophilic interactions is not sufficient for emissive behaviour and that unsupported intermolecular aurophilic interactions are required. The shortest intermolecular Au–Au distance is 7.4798(1) Å, at 100 K, which is clearly outside the range where interactions are significant.

Having investigated the effect of changing temperature on the luminescence properties of these four complexes, we then embarked upon a series of high pressure single crystal X-ray diffraction studies that were performed at the Advanced Light Source, Berkeley, California. Pressure was applied using a Merill–Basett diamond anvil cell using 4:1 methanol/ethanol as the hydrostatic medium and ruby fluorescence as the pressure calibrant on the four compounds. Compounds **1** and **2** were studied from ambient up to 7.80 and 5.1 GPa, respectively, while **4** was investigated up to 2.31 GPa before a phase transition was observed resulting in a loss of Bragg peaks in the diffraction images (see the Supporting Information). Data were collected on **3** up to 0.17 GPa before it underwent a phase transformation and loss of crystallinity (see the Supporting Information).

All four compounds studied undergo contraction as pressure is increased with remarkable similarities between the behaviour of the materials with temperature and pressure.

However, it is likely that in terms of thermodynamics, entropy is the driving force in the variable temperature studies while in the high-pressure studies, the volume reduction can be correlated to pressure. In this case the two effects have similar results. Analysis of the bulk modulus (*B*_0_) taken from a third-order Birch–Murnaghan equation of state^15^ fitted to **1**, **2** and **4** (Table 3, Table 4, Table 5), suggests that **4** is the most compressible followed by **2** and finally **1**.

**Table 3:** Equation of state parameters from high pressure data.

	Equation of state parameters		
	*V*_0_ [Å^3^]	*B*_0_ [GPa]	*B*′
1	1265.9(6)	9.20(1.2)	10.23(1.2)
2	2168.2(1)	6.42(1.3)	11.90(2.6)
4	5748.5(2)	6.04 (1.5)	12.94(2.4)

**Table 4:** Unit cell volume and void volume decrease with increasing pressure for compounds 1 and 2.

Pressure	Parameters (compound 1)					Pressure	Parameters (compound 2)				
[GPa]	*a*	*b*	*c*	*β*	*V*	[GPa]	*a*	*b*	*c*	*β*	*V*
0.00^[a]^	8.3340(12)	13.919(3)	10.829(2)	106.890(2)	1265.9(3)	0.00^[a]^	8.9753(2)	22.3828(6)	10.8274(3)	94.550(3)	2168.29(10)
1.04	8.1046(16)	13.543(3)	10.700(2)	107.27(3)	1166.4(4)	0.65	8.7372(18)	22.018(4)	10.483(2)	93.08(3)	2013.8(7)
2.30	7.9763(17)	13.295(3)	10.626(2)	107.31(3)	1103.4(4)	1.11	8.6507(17)	21.908(4)	10.356(2)	92.52(3)	1960.7(7)
3.40	7.9044(16)	13.295(3)	10.626(2)	107.26(3)	1066.4(4)	1.59	8.5716(17)	21.805(4)	10.237(2)	92.00(3)	1912.1(7)
3.80	7.8837(16)	13.217(3)	10.606(2)	107.21(3)	1055.7(4)	2.35	8.4772(17)	21.686(4)	10.062(2)	91.40(3)	1849.2(6)
5.04	7.8073(16)	13.026(3)	10.538(2)	107.26(3)	1023.4(4)	3.14	8.4092(17)	21.573(4)	9.913(2)	90.60(3)	1798.2(6)
6.22	7.7527(16)	12.866(3)	10.491(2)	107.21(3)	999.6(4)	3.91	8.3405(17)	21.498(4)	9.765(2)	90.21(3)	1750.9(6)
7.80	7.6803(15)	12.717(3)	10.442(2)	107.23(3)	974.2(3)	5.18	8.289(5)	21.438(5)	9.569(5)	89.582(5)	1700.4(14)

[a] ambient pressure.

**Table 5:** Unit-cell volume and void volume decrease with increasing pressure for compounds 3 and 4.

Pressure	Parameters (compound 3)				Pressure	Parameters (compound 4)		
[GPa]	*a*	*b*	*c*	*V*	[GPa]	*a/b*	*c*	*V*
0.00^[a]^	7.4065(10)	16.001(2)	26.502(3)	3140.9(7)	0.00^[a]^	16.0783(3)	25.6914(7)	5751.7(2)
1.04	7.2885(15)	15.843(3)	26.141(5)	3018.5(11)	0.52	15.8344(7)	25.013(2)	5431.3(6)
					0.97	15.7110(9)	24.560(3)	5250.0(7)
					1.24	15.6768(7)	24.379(2)	5188.8(6)
					1.31	15.6632(7)	24.327(2)	5168.7(6)
					1.76	15.5942(7)	24.002(2)	5054.9(5)
					2.05	15.5591(7)	23.825(2)	4994.9(5)
					2.31	15.5377(7)	23.726(2)	4960.5(5)

[a] ambient pressure.

The values are typical for a molecular crystal when compared to other examples in the literature and similar to other values given for organometallic complexes.^16^ As expected much larger changes in unit-cell volume are observed with changes in pressure than with temperature due to the ease with which relative high pressures may be achieved within the diamond anvil cells; for example, a 2.5 % change in volume observed between 100–293 K for **1** compared to a 22.6 % change between ambient and 7.80 GPa. Computational studies on the high-pressure structures of **1** demonstrated reasonable correlation with the experimentally observed compressibily; a 0 K value of 7.21 GPa (*B*′ 8.5) was calculated from density functional perturbation theory. Compression occurs in a similar manner to that observed with temperature, reducing intertrimer distance and therefore intermolecular aurophilic interaction distance in compounds **1**–**3** (Table 6).

**Table 6:** Intermolecular interactions with pressure for compounds 1–3.

Pressure	Parameters (compound1)			Pressure	Parameters (compound2)			Pressure	Parameters (compound3)		
[GPa]	Au1–Au1′	Au1–Au2′	Au1–Au3′	[GPa]	Au1–Au2′	Au2–Au3′	Au3–Au1′	[GPa]	Au1–Au1′	Au2–Au3′	Au2–Au3′
0.00^[a]^	3.7346(9)	4.4924(8)	3.3095(7)	0.00^[a]^	3.3345(4)	4.7791(5)	5.1049(5)	0.00^[a]^	3.7043(5)	3.6782(7)	3.854(7)
1.04	3.6357(18)	4.3371(9)	3.1890(7)	0.65	3.2342(11)	4.6269(13)	5.0007(18)	0.17	3.6444(8)	3.6267(10)	3.7997(10)
2.30	3.586(2)	4.2164(9)	3.0997(8)	1.11	3.1992(11)	4.5686(13)	4.9673(17)				
3.40	3.559(3)	4.1413(10)	3.0491(8)	1.59	3.1676(12)	4.5167(14)	4.9378(19)				
3.80	3.555(2)	4.1170(8)	3.0342(7)	2.35	3.128(2)	4.4421(13)	4.91(2)				
5.04	3.522(2)	4.0581(9)	2.9934(7)	3.14	3.098(15)	4.3745(19)	4.885(3)				
6.22	3.499(2)	4.0105(8)	2.9631(7)	3.91	3.0787(19)	4.3142(11)	4.8703(17)				
7.80	3.465(2)	3.9650(9)	2.9340(8)	5.18	3.055(3)	4.224(2)	4.846(3)				

[a] ambient pressure.

With the larger changes in volume observed with pressure increase, larger changes in intermolecular Au⋅⋅⋅Au distances in **1** and **2** are also observed, with a shortening of up to 0.3 Å, and large enough to induce large spectroscopic changes. High pressure computational work once again provided good correlation between the experimentally observed compressibilty of aurophilic interactions and that observed in computational simulation on the prototype system of compound **1** (see the Supporting Information).

Other steric interactions within the structure become of interest at higher pressures. The H⋅⋅⋅H steric interactions observed between aurophilically bound trimers in **1** and **2** become progressively more hindered as the hydrogen atoms are forced to occupy a smaller volume. The use of Hirshfeld surfaces is an excellent tool for quantifying important solid-state interactions^17^ and has been used extensively in the analysis of organic and organometallic systems under a variety of conditions.^18^ Parsons et al. have studied organic compounds at high pressure extensively^19^ and have concluded that the (*D*_i_+*D*_e_) value of steric H⋅⋅⋅H may be used to suggest potential phase transitions, with a value of 1.7 Å being the limit in small organics before a transition is observed.^19^ Analysis of the steric interactions of **2** reveals that at 5.31 GPa a value of 1.6 Å (*D*_i_+*D*_e_) is observed, making these H⋅⋅⋅H interactions some of the shortest in the literature (see the Supporting Information), with the caveat that for these structure refinements the hydrogen atoms are constrained to ride on the relevant carbon atoms, and are not refined freely.

Since the increase in aurophilic interactions contribute to the arrangement of molecules within the crystal, and thus the formation of the short H⋅⋅⋅H contacts, it is possible that aurophilic interactions could be used to create sterically congested molecules in the solid state that may display unique phase behaviour.

**Figure 4:**
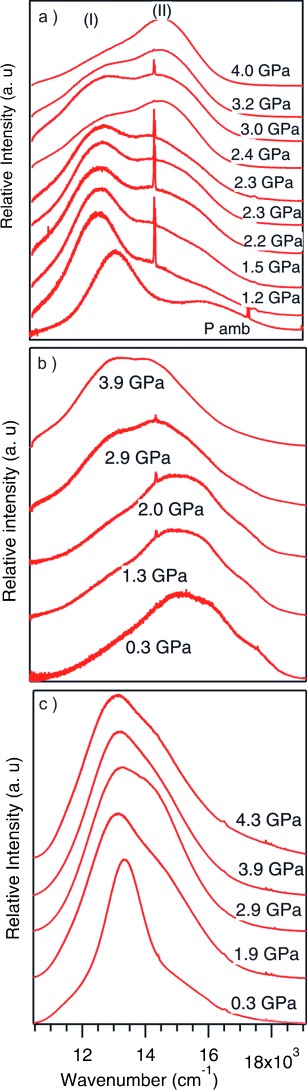
High pressure emission spectra for: a) 1, b) 2, and c) 3.

High pressure luminescence spectroscopy was performed on all compounds. Once again no luminescence was observed upon compression of compound **4**, as expected, however, all other compounds displayed dramatic changes in their emission spectra. For compound **1** the initial spectra display a broad band at approximately 13 200 cm^−1^ (I) with a shoulder at about 16 000 cm^−1^ (II). The *E*_max_ of the broad peak shifts by −360 cm^−1^ per GPa between ambient and 1.54 GPa. We note that the width at half maximum is again about 3700 cm^−1^ (Figure 4 a). At intermediate temperature and pressure, the spectrum of **1** displays two bands. The lower energy band position and intensity is more sensitive to pressure and temperature variation, while the higher energy band appears to be relatively insensitive to external perturbation.

**Figure 5:**
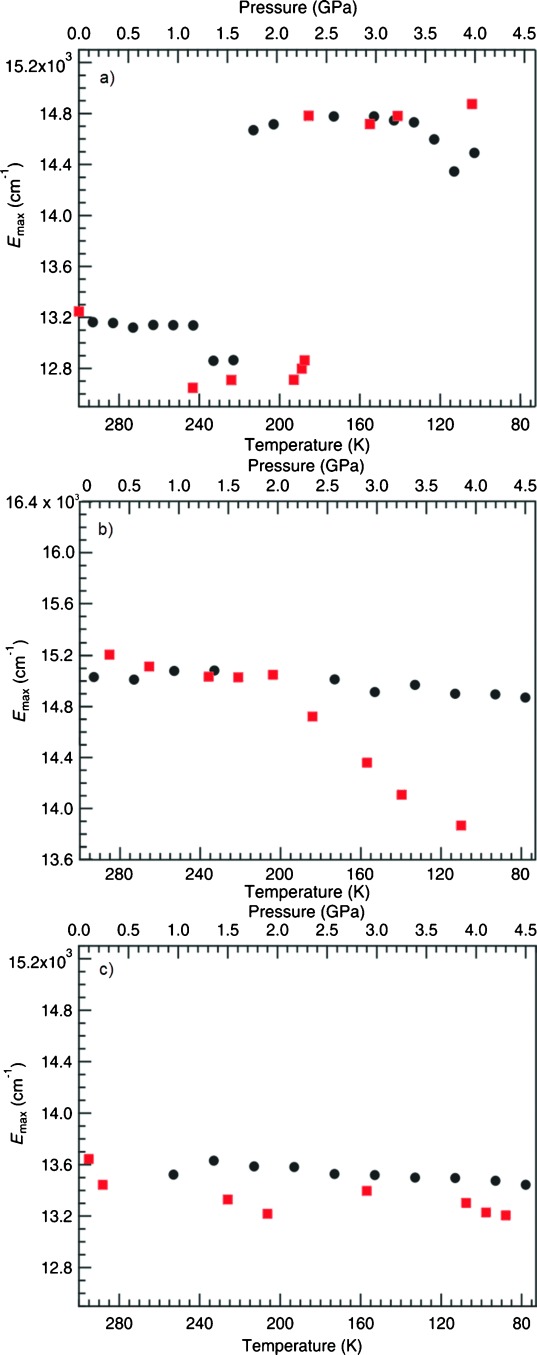
Comparison of variable pressure (red) and variable temperature (black) emission energies for: a) 1, b) 2, and c) 3. Error bars are smaller than the size of the circles and squares.

Compound **2** behaves more typically, displaying a red shift from approximately 15 200 cm^−1^ to about 13 870 cm^−1^ by about 400 cm^−1^ per GPa (Figure 4 b), in agreement with the prediction that reducing the aurophilic interaction length by 0.258(2) Å from ambient to 3.91 GPa results in a red shift that may be attributed to a reduction in the HOMO–LUMO gap in the complex.^11^ The width at half maximum is about 3700 cm^−1^, which is similar to that observed in **1**. The variation of the *E*_max_ of the emission for temperature and pressure is similar for both parameters until 2 GPa or 200 K when the reduction of the intermolecular distance is more significant with pressure than with temperature, resulting in a pronounced red shift which varies linearly as 630 cm^−1^ per GPa in the 1.95–3.85 GPa region (Figure 5 b).

Compound **3** displays luminescence after minimal compression; with an emission at approximately 13 340 cm^−1^ that red shifts to a small degree between ambient and 4.3 GPa (Figure 4 c). Comparison between the temperature and pressure data demonstrate a similar variation in *E*_max_ over the range studied (Figure 5 c).

For compounds **2** and **3** there is a reasonable correlation between the behaviour of the complexes at high pressure and low temperature both crystallographically and spectroscopically. On compression or contraction of intermolecular aurophilic bonds, observed by diffraction, there are corresponding significant shifts in emission wavelength that can be understood with theoretical calculations.

Compound **1** is of the greatest interest due to its unusual luminescent behaviour at high pressure and low temperature. Analysis of the computational work performed suggests that a gradual blue shift should be observed with decreasing temperature and a gradual but large red shift in the emission should occur with increasing pressure in all the systems studied. It is apparent in both the variable temperature and high pressure spectra of **1** that a new band begins to dominate the emission spectra. The wavelength shift with increasing temperature and increasing pressure is remarkably similar in both cases and in both occur quite suddenly rather than gradually (213–223 K and 1.50–1.70 GPa; Figure 5 a). The transition coincides with changes in the variable temperature and high pressure Raman spectroscopy in the regions associated with Au–N and Au–Au interactions although no significant events such as a phase transition are observed by crystallographic means.

**Figure 6:**
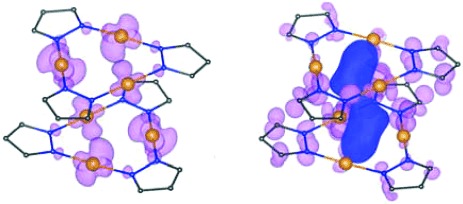
Highest occupied and lowest unoccupied crystal orbitals (HOCO and LUCO, respectively) for 1. Blue lobe in the LUCO indicates the large intertrimer orbital.

The DFT calculations performed on **1**, based on the crystallographically determined structures, indicate the nature of the highest occupied (HOCO) and lowest unoccupied (LUCO) crystal orbitals (Figure 6). The presence of the large intertrimer LUCO orbital points to the origin of the low-energy transition in the crystal as being due to the aurophilic interactions.^11^, ^20^ Analysis of the optical band gap variation with temperature and pressure variations identifies a direct correlation with the Au–Au distance, as expected. A red shift is found with increasing pressure (shorter Au–Au separations), while a weaker blue shift is found with increasing temperatures (longer Au–Au distances). Hence, a corresponding increase of 0.1 eV in the band gap is observed from 100 to 300 K. An increase of the pressure from ambient to 7.8 GPa produces a decrease of the band gap of 0.66 eV. As such the DFT calculations explain the behaviour of the individual bands upon cooling and pressurisation; however, the calculations do not explain the appearance and evolution of intensity of the two bands, which would require explicit excited-state techniques.

Luminescence is an effect often dominated by minority sites where excitation energy accumulates through energy transfer, in particular in concentrated molecular crystals. In contrast, X-ray diffraction only characterises the perfectly crystalline parts of the sample. This is an aspect to be kept in mind when comparing structures and spectra, in particular at high pressure, where more defects are created in the crystals, and may explain the observations here.

## Conclusion

For complexes **1**–**4** at high pressure and low temperature the combined spectroscopic and crystallographic results show that subtle structural effects can lead to drastic changes of the luminescence spectra, likely to be due to energetically close, but different electronic states.

For compounds **2** and **3**, as in other solids with aurophilic interactions a significant shift of the luminescence band maximum to lower energy occurs with increasing pressure, and a good correlation between the effects of high temperature and low pressure.10a, c Compound **1**, however, shows very different behaviour with a new band in the spectrum being observed between ambient pressure and 1.54 GPa. At pressures higher than 2 GPa, this higher-energy band at 16 000 cm^−1^ becomes the most intense feature of the spectrum. The transition is continuous, and hence not indicative of a structural phase transition. Raman spectra also do not show sudden changes, again not providing evidence for an abrupt phase transition.

Significant changes of the spectrum are usually associated to a new band at lower energy, due to emissive traps sensitised by energy transfer induced by degradation of the crystal structure as pressure increases.^11^ In compound **1**, it appears that the electronic structure changes with increasing pressure or decreasing temperature, leading to a different emitting state as aurophilic distances decrease.

In these systematic studies we have shown that temperature and pressure are both effective tools for varying the wavelength of luminescent emission in compounds where intermolecular aurophilic interactions are present. The study highlights the importance of direct correlation between structural and spectroscopic data to interpret the results. The unusual behaviour observed in **1** demonstrates that even detailed structural analysis may not provide a full explanation of the spectroscopic observations. Focus should be placed on the development of techniques that are capable of making simultaneous high pressure crystallographic and spectroscopic measurements to probe the dynamics associated with emissive phenomena.

## Experimental Section

All reagents were used as received from commercial suppliers. 3,4,5-Trimethylpyrazole and [Au(tht)Cl] (tht=tetrahydrothiophene) were synthesised following a literature procedure.^21^ The synthesis of all trimers was based on the synthetic procedure for tris(μ2-pyrazolato-*N*,*N*′)-tri-gold(I) given below [tris(μ2-pyrazolato-*N*,*N*′)-tri-gold(I)]. Synthesis of [Au_3_(pyra)_3_] was carried out following a method similar to a literature procedure.3a 1*H*-Pryazole (0.200 mmol; 0.014 g) and [Au(tht)Cl] (0.1 mmol; 0.032 g) were dissolved in THF (2 mL) in a vial. The vial was placed in a larger air-tight container. NEt_3_ (0.1 mmol; 0.014 mL) was added to THF (5 mL) and added to the larger container. The container was sealed and placed in a fridge and left for one month to yield crystals of two morphologies, fine needles and large blocks. The product was collected and washed with DCM (5 mL) and the remaining crystals were isolated to yield the desired product suitable for X-ray analysis (yield=41 %; 0.0107 g). Bulk purity of sample and phase were determined by powder X-ray diffraction due to insolubility of the compound in all NMR solvents. CHN analysis, predicted: 13.65, 1.15, 10.61; observed: 13.65, 1.19, 10.58, respectively.

### [Tris(μ2-3,4,5-trimethylpyrazolato-*N*,*N*′)-tri-gold(I)]

3,4,5-Trimethylpyrazole (0.16 mmol, 0.0175 g) and [Au(tht)Cl] (0.16 mmol, 0.05 g) were added to THF (10 mL) in a vial. The vial was then placed inside a larger vial with NEt_3_ (0.16 mmol, 0.022 mL) in THF (10 mL). The container vial was sealed and left for 1 month to yield very small crystals. The crystals were of insufficient quality for X-ray analysis and were therefore regrown from hot fluorobenzene, and left to cool from 90 °C to room temperature in a React array microvate parallel synthesiser crystallisation apparatus over the course of two days to yield tiny block crystals suitable for X-ray analysis. CHN analysis predicted: 23.54, 2.96, 9.15; observed: 23.51, 2.85, 9.11, respectively.

3-Methyl-5-phenyl-1*H*-pyrazole phenylbutane-1,3-dione (0.12 mol; 19.26 g) was suspended in EtOH. Hydrazine monohydrate (0.15 mol; 7.5 mL) was added dropwise under stirring, resulting in a clear, yellowish solution. The reaction was refluxed for 30 min and cooled to RT. A voluminous, cotton-wool-like solid formed, H_2_O (150 mL) was added, filtered and washed with more H_2_O. The product was dried in vacuo with gentle heating to remove all remaining H_2_O and hydrazine to yield a white non-crystalline solid (Yield=76.8 %; 14.56 g). ^1^H NMR (400 Hz, CDCl_3_): *δ*=11.00 (broad s; 1 H), 7.71 (d, 2 H), 7.32 (m, 3 H), 6.34 (d, 1 H), 2.27 ppm (d, 3 H); ^13^C NMR pendant (500 Hz, CDCl_3_): *δ*=227.18 (C), 223.33 (CH), 222.47 (CH), 220.48 (CH), 197.2 (CH), 106.3 ppm (CH3).

### [Tris(μ2-3-methyl-5-phenylpyrazolato-*N*,*N*′)-tri-gold(I)]

This was synthesised based on a literature method. 3-Methyl-5-phenyl-1*H*-pyrazole (0.156 mmol; 0.025 g) and [Au(tht)Cl] (0.156 mmol; 0.05 g) were dissolved in THF (10 mL). NEt_3_ (0.156 mmol; 0.022 mL) was added to the solution. Small crystals precipitated from the solution immediately and were filtered from the solution. The solution was left to evaporate in a test tube over the course of several weeks to yield crystals suitable for X-ray analysis (Yield=54.0 %; 0.0298 g). Bulk purity of sample and phase were determined by powder X-ray diffraction. CHN analysis predicted: 33.91, 2.56, 7.91; observed: 34.14, 2.82, 7.40, respectively.

### [Tris(μ2–3,5-diphenylpyrazolato-*N*,*N*′)-tri-gold(I)]

3,5-Diphenyl-1*H*-pyrazole (0.624 mmol; 0.137 g) and [Au(tht)Cl] (0.624 mmol; 0.200 g) were dissolved in THF (20 mL). NEt_3_ (0.172 mmol; 0.087 mL) was added to the solution. Small crystals precipitated from the solution immediately. The solution was stirred for 6 h, filtered and the solvent removed in vacuo to yield an off white powder. Crystallisation from THF yielded crystals suitable for X-ray analysis (yield=18.69 %; 0.1456 g). Bulk purity of sample and phase were determined by powder X-ray diffraction. CHN analysis predicted: 43.38, 2.66, 6.73; observed: 43.19, 2.72, 6.61, respectively.

### Crystallographic measurements

Variable temperature single crystal X-ray diffraction experiments were performed on either an Oxford Diffraction Gemini A Ultra diffractometer at the University of Bath equipped with an Oxford Instruments Cryojet XL crystal cooling device, or a Bruker APEX II CCD diffractometer equipped with an Oxford Cryosystems cryostream 700 series crystal cooling device (Station 11.3.1, Advanced Light Source, Lawrence Berkeley National Laboratory, US). Data were collected and processed using CrysalisPro and APEX II. Structure solution was done using SHELX-86 and refinement was performed using SHELX-97.^22^ Features of WinGX,^23^ Crystals,^24^ Olex-2,^25^ Xseed,^26^ Mercury and Crystal Explorer were all used in the data analysis.^13^, ^27^ EOSFit and PASCal were also used.^14^, ^28^

High-pressure single crystal X-ray diffraction experiments were performed on a 3-circle Bruker APEX II CCD diffractometer at station 11.3.1, Advanced Light Source, Lawrence Berkeley National Laboratory, US. Compounds with crystallographic symmetry lower than orthorhombic were collected in multiple cell orientations to increase data completeness. A Merrill–Bassett diamond anvil cell was used for the high-pressure measurements using boehler–almax diamonds with 600 μm culets. Laser cut tungsten or steel (200 μm thickness) was used as the gasket material. Gasket holes were drilled using an Oxford Lasers laser mill to 200 μm diameter unless otherwise stated. Loading of the cell for all samples was performed using 4:1 methanol/ethanol mix as a hydrostatic medium, using ruby powder as the pressure callibrant and pressure calibration was performed via the Ruby fluorescence method.^29^ High pressure data were integrated using the APEX2 software suite. Shielding of the diffraction pattern by the DAC was dealt with by the generation of dynamic masks using an external program.^30^ Datasets were merged using XPREP and a multiscan absorption correction was performed using SADABS.^31^ Data were refined against a previously determined room temperature structure by full-matrix least squares on *F*^*2*^ using SHELXL-97.^22^ All C–C or C–N distances in the structure were restrained to the values of the room temperature structure, on the assumption that such interactions are relatively resilient to compression. Metal–metal interactions or metal–C or –N interactions were freely refined.

CCDC 971102 (**1**, 293 K), 971101 (**1**, 270 K), 971100 (**1**, 240 K), 971099 (**1**, 210 K), 971098 (**1**, 180 K), 971097 (**1**, 150 K), 971096 (**1**, 120 K), 971095 (**1**, 100 K), 971088 (**1**, 1.04 GPa), 971089 (**1**, 2.30 GPa), 971090 (**1**, 3.40 GPa), 971091 (**1**, 3.80 GPa), 971092 (**1**, 5.04 GPa), 971093 (**1**, 6.22 GPa), 971094 (**1**, 7.80 GPa), 971128 (**2**, 293 K), 971126 (**2**, 270 K), 971124 (**2**, 240 K), 971123 (**2**, 210 K), 971122 (**2**, 180 K), 971121 (**2**, 150 K), 971120 (**2**, 120 K), 971119 (**2**, 100 K), 971081 (**2**, 0.65 GPa), 971082 (**2**, 1.11 GPa), 971083 (**2**, 1.59 GPa), 971084 (**2**, 2.35 GPa), 971085 (**2**, 3.14 GPa), 971086 (**2**, 3.91 GPa), 971087 (**2**, 5.18 GPa), 971111 (**3**, 293 K), 971112 (**3**, 270 K), 971113 (**3**, 240 K), 971114 (**3**, 210 K), 971115 (**3**, 180 K), 971116 (**3**, 150 K), 971117 (**3**, 120 K), 971118 (**3**, 100 K), 971071 (**3**, ambient), 971072 (**3**, 0.17 GPa), 971110 (**4**, 293 K), 971109 (**4**, 270 K), 971108 (**4**, 240 K), 971107 (**4**, 210 K), 971106 (**4**, 180 K), 971105 (**4**, 150 K), 971104 (**4**, 120 K), 971103 (**4**, 100 K), 971073 (**4**, 0.52 GPa), 971074 (**4**, 0.97 GPa), 971075 (**4**, 1.24 GPa), 971076 (**4**, 1.31 GPa), 971077 (**4**, 1.76 GPa), 971078 (**4**, 2.05 GPa), 971079 (**4**, 2.31 GPa) contain the supplementary crystallographic data for this paper. These may be obtained free of charge from The Cambridge Crystallographic Data Centre via www.ccdc.cam.ac.uk/data_request/cif.

### Spectroscopic measurements

Raman and luminescence spectra were measured with a Renishaw Invia Raman imaging microscope equipped with a Peltier-cooled CCD camera. Excitation sources were a 488 nm argon ion laser and 514 nm diode laser for the luminescence experiments and a 785 nm diode laser for the Raman experiments. The microscope was used to focus light onto a spot of approximately 1 μm in diameter and to collect the scattered light. Low-temperature Raman experiments were obtained by coupling a Linkam coldfinger cryostat to the apparatus with liquid nitrogen used as coolant. A Janis closed cycle helium cryostat was used for low-temperature luminescence experiments. Pressure-dependent measurements on solid samples in nujol were made with a diamond-anvil cell (DAC, High-Pressure Diamond Optics). The ruby R1 line method12 was used to calibrate the hydrostatic pressure inside the gasketed cell. All pressure-induced phenomena reported here are reversible upon gradual release of external pressure.

### Computational details

Calculations of the periodic solids were performed at the level of DFT within the plane-wave pseusodpotential code VASP. Exchange-correlation effects were treated using the semi-local PBE functional revised for solids (PBEsol). A kinetic energy cut-off of 600 eV was used to construct the electronic basis set, with a k-point density of 4×2×2 to sample the first Brillouin zone. For Au, the 5d106s1 electrons were explicitly treated as valence, with scalar relativistic effects included. The 166 atom unit cell (432 valence electrons) of compound **1** was modelled. The structural coordinates were optimised to within 0.01 eV Å^−1^ under the space group symmetry and with the lattice vectors held at the (temperature and pressure dependent) values determined from the single crystal diffraction measurements.

## Supporting Information

As a service to our authors and readers, this journal provides supporting information supplied by the authors. Such materials are peer reviewed and may be re-organized for online delivery, but are not copy-edited or typeset. Technical support issues arising from supporting information (other than missing files) should be addressed to the authors.

miscellaneous_information

## References

[1a] Yam VW-W, Cheng EC-C (2008). Chem. Soc. Rev.

[1b] Yam VW-W, Cheng EC-C, Balzani V, Campagna S (2007). Photochemistry and Photophysics of Coordination Compounds II.

[1c] Schmidbaur H (2000). Gold Bull.

[1d] Tiekink ERT (2014). Coord. Chem. Rev.

[2] Yang C, Messerschmidt M, Coppens P, Omary MA (2006). Inorg. Chem.

[3a] Yang G, Raptis RG (2003). Inorg. Chem.

[3b] Vickery JC, Olmstead MM, Fung EY, Balch AL (1997). Angew. Chem. Int. Ed. Engl.

[3c] Fung EY, Olmstead MM, Vickery JC, Balch AL (1998). Coord. Chem. Rev.

[3d] White-Morris RL, Olmstead MM, Attar S, Balch AL (2005). Inorg. Chem.

[3e] Gussenhoven EM, Fettinger JC, Pham DM, Malwitz MM, Balch AL (2005). J. Am. Chem. Soc.

[3f] Olmstead MM, Jiang FL, Attar S, Balch AL (2001). J. Am. Chem. Soc.

[3g] Hayashi A, Olmstead MM, Attar S, Balch AL (2002). J. Am. Chem. Soc.

[3h] Kim SJ, Kang SH, Park KM, Kim H, Zin WC, Choi MG, Kim K (1998). Chem. Mater.

[3i] Omary MA, Rawashdeh-Omary MA, Gonser MWA, Elbjeirami O, Grimes T, Cundari TR (2005). Inorg. Chem.

[3j] Yang G, Martinez JR, Raptis RG (2009). Inorg. Chim. Acta.

[3k] Kishimura A, Yamashita T, Aida T (2005). J. Am. Chem. Soc.

[3l] Yang G, Raptis RG (2002). Dalton Trans.

[3m] Burini A, Fackler JP, Galassi R, Pietroni BR, Staples RJ (1998). Chem. Commun.

[3n] Elbjeirami O, Rashdan MD, Nesterov V, Rawashdeh-Omary MA (2010). Dalton Trans.

[4] Mendizabal F, Aguilera B, Olea-Azar C (2007). Chem. Phys. Lett.

[5] Burini A, Bravi R, Fackler JP, Galassi R, Grant TA, Omary MA, Pietroni BR, Staples RJ (2000). Inorg. Chem.

[6] Vorontsov II, Kovalevsky AY, Chen YS, Graber T, Gembicky M, Novozhilova IV, Omary MA, Coppens P (2005). Phys. Rev. Lett.

[7a] Byrne PJ, Richardson PJ, Chang J, Kusmartseva AF, Allan DR, Jones AC, Kamenev KV, Tasker PA, Parsons S (2012). Chem. Eur. J.

[7b] Fairen-Jimenez D, Moggach SA, Wharmby MT, Wright PA, Parsons S, Duren T (2011). J. Am. Chem. Soc.

[7c] Prescimone A, Milios CJ, Moggach S, Warren JE, Lennie AR, Sanchez-Benitez J, Kamenev K, Bircher R, Murrie M, Parsons S, Brechin EK (2008). Angew. Chem. Int. Ed.

[7d] Wong HLS, Allan DR, Champness NR, McMaster J, Schroeder M, Blake AJ (2013). Angew. Chem. Int. Ed.

[7e] Li W, Probert MR, Kosa M, Bennett TD, Thirumurugan A, Burwood RP, Parinello M, Howard JAK, Cheetham AK (2012). J. Am. Chem. Soc.

[8a] Cairns AB, Catafesta J, Levelut C, Rouquette J, van der Lee A, Peters L, Thompson AL, Dmitriev V, Haines J, Goodwin AL (2013). Nat. Mater.

[8b] Woodall CH, Beavers CM, Christensen J, Hatcher LE, Intissar M, Parlett A, Teat SJ, Reber C, Raithby PR (2013). Angew. Chem. Int. Ed.

[9a] Okano Y, Zhou B, Tanaka H, Adachi T, Ohishi Y, Takata M, Aoyagi S, Nishibori E, Sakata M, Kobayashi A (2009). J. Am. Chem. Soc.

[9b] Yzambart G, Bellec N, Nasser G, Jeannin O, Roisnel T, Fourmigue M, Auban-Senzier P, Iniguez J, Canadell E, Lorcy D (2012). J. Am. Chem. Soc.

[10a] Strasser J, Yersin H, Patterson HH (1998). Chem. Phys. Lett.

[10b] Fischer P, Mesot J, Lucas B, Ludi A, Patterson H, Hewat A (1997). Inorg. Chem.

[10c] Yersin H, Ulrich R (1995). Inorg. Chem.

[11] Baril-Robert F, Radtke MA, Reber C (2012). J. Phys. Chem. C.

[12] Murray HH, Raptis RG, Fackler JP (1988). Inorg. Chem.

[13] Macrae CF, Bruno IJ, Chisholm JA, Edgington PR, McCabe P, Pidcock E, Rodriguez-Monge L, Taylor R, van de Streek J, Wood PA (2008). J. Appl. Crystallogr.

[14] Cliffe MJ, Goodwin AL (2012). J. Appl. Crystallogr.

[15a] Birch F (1947). Phys. Rev.

[15b] Angel R (2002).

[16] Slebodnick C, Zhao J, Angel R, Hanson BE, Song Y, Liu ZX, Hemley RJ (2004). Inorg. Chem.

[17] Spackman MA, Jayatilaka D (2009). CrystEngComm.

[18a] Casati N, Macchi P, Sironi A (2009). Chem. Eur. J.

[18b] Collins A, Wilson CC, Gilmore CJ (2010). CrystEngComm.

[18c] Durka K, Hoser AA, Kaminski R, Lulinski S, Serwatowski J, Kozminski W, Wozniak K (2011). Cryst. Growth Des.

[18d] Fabbiani FPA, Allan DR, Parsons S, Pulham CR (2006). Acta Crystallogr. Sect. B.

[18e] Fabbiani FPA, Levendis DC, Buth G, Kuhs WF, Shankland N, Sowa H (2010). CrystEngComm.

[18f] Johnstone RDL, Lennie AR, Parker SF, Parsons S, Pidcock E, Richardson PR, Warren JE, Wood PA (2010). CrystEngComm.

[18g] Loots L, Barbour LJ (2012). CrystEngComm.

[18h] McKinnon JJ, Fabbiani FPA, Spackman MA (2007). Cryst. Growth Des.

[18i] McKinnon JJ, Jayatilaka D, Spackman MA (2007). Chem. Commun.

[18j] McKinnon JJ, Mitchell AS, Spackman MA (1998). Chem. Eur. J.

[18k] McKinnon JJ, Spackman MA, Mitchell AS (2004). Acta Crystallogr. Sect. B.

[18l] Moggach SA, Marshall WG, Parsons S (2006). Acta Crystallogr. Sect. B.

[18m] Moggach SA, Parsons S, Wood PA (2008). Crystallogr. Rev.

[18n] Parkin A, Barr G, Dong W, Gilmore CJ, Jayatilaka D, McKinnon JJ, Spackman MA, Wilson CC (2007). CrystEngComm.

[18o] Spackman MA, Byrom PG (1997). Chem. Phys. Lett.

[18p] Spackman MA, McKinnon JJ (2002). CrystEngComm.

[18q] Wood PA, Haynes DA, Lennie AR, Motherwell WDS, Parsons S, Pidcock E, Warren JE (2008). Cryst. Growth Des.

[18r] Shepherd HJ, Rosa P, Fallis IA, Guionneau P, Howard JAK, Goeta AE (2012). J. Phys. Chem. Solids.

[19] Wood PA, McKinnon JJ, Parsons S, Pidcock E, Spackman MA (2008). CrystEngComm.

[20] Zhang MX, Mang CY, Wu KC (2006). J. Mol. Struct.

[21] Uson R, Laguna A, Laguna M, Briggs DA, Murray HH, Fackler JP (1989). Inorg. Synth.

[22] Sheldrick GM (2008). Acta Crystallogr. Sect. A.

[23a] Farrugia LJ (1999). J. Appl. Crystallogr.

[23b] Farrugia LJ (2012). J. Appl. Crystallogr.

[24] Betteridge PW, Carruthers JR, Cooper RI, Prout K, Watkin D (2003). J. Appl. Crystallogr.

[25] Dolomanov OV, Bourhis LJ, Gildea RJ, Howard JAK, Puschmann H (2009). J. Appl. Crystallogr.

[26] Barbour LJ (2001). J. Supramol. Chem.

[27a] Wolff SK, Grimwood DJ, McKinnon JJ, Turner MJ, Jayatilaka D, Spackman MA (2010).

[27b] Wolff SK, Grimwood DJ, McKinnon JJ, Turner MJ, Jayatilaka D, Spackman MA (2012).

[28] Angel R (2000). Rev. Mineral. Geochem.

[29] Piermarini GJ, Block S, Barnett JD, Forman RA (1975). J. Appl. Phys.

[30] Dawson A, Allan DR, Parsons S, Ruf M (2004). J. Appl. Crystallogr.

[31] SADABS (2005).

