# Supplementary material for: Tunable Trimers: Using Temperature and Pressure to Control Luminescent Emission in Gold(I) Pyrazolate-Based Trimers
Source: Chemistry. 2014 Oct 21;20(51):16933–42. doi: 10.1002/chem.201404058 (PMC4288234; doi:10.1002/chem.201404058)
Supplement: Supplementary file 1 — miscellaneous_information [file chem0020-16933-sd1.pdf]

CHEMISTRY
A European Journal

Supporting Information

© Copyright Wiley-VCH Verlag GmbH & Co. KGaA, 69451 Weinheim, 2014

Tunable Trimers: Using Temperature and Pressure to Control Luminescent Emission in Gold(I) Pyrazolate-Based Trimers

Christopher H. Woodall,[a] Sara Fuertes,[a] Christine M. Beavers,[b] Lauren E. Hatcher,[a] Andrew Parlett,[a] Helena J. Shepherd,[a] Jeppe Christensen,[a] Simon J. Teat,[b] Mourad Intissar,[c] Alexandre Rodrigue-Witchel,[c] Yan Suffren,[c] Christian Reber,*[c] Christopher H. Hendon,[a] Davide Tiana,[a] Aron Walsh,[a] and Paul R. Raithby*[a]

chem_201404058_sm_miscellaneous_information.pdf

CONTENTS

Synthetic details 2

Void details for compounds 3 and 4 2

Void analysis with temperature 4

Void analysis with pressure 4

Relation of thermal indicatrix to unit cell content 5

Equation of State Details 6

Calculated axes of compression and contraction 7

Unusual crystallographic behaviour of compound 3 9

Unusual crystallographic behaviour of compound 4 10

Analysis of non-aurophilic interactions with pressure 12

Raman Spectroscopy 14

Computational band-gap changes 15

Crystallography tables 15

References 24

Synthetic details

All synthetic procedures involving the handling and preparation of air water sensitive reagents were performed using standard Schlenk techniques under an atmosphere of purified nitrogen. All solvents involved in the handling of air or water sensitive species were dried prior to use using an automated solvent purification system. All chemicals were purchased from a commercial source and used without further purification unless stated.

Void details for compounds 3 and 4

Compounds **3** and **4** possess voids of different sizes within their crystal structures as calculated using Mercury.[1] The term crystal structure void is used to define areas within a crystal structure that are not filled with atoms that make up part of the asymmetric unit of a crystal structure. Compound **4** possess a small solvent inaccessible void approximately 131 Ǻ^3 representing 2.3 % of the total volume of the structure at room temperature. The void is present half way between gold (I) trimer molecules as they stack down the *c* axis and is surrounded by six phenyl groups from different molecules in the unit cell, the rings turning to be parallel to the opposing phenyl ring around the void, shown in Figure 1. The voids appear to be completely isolated from one another and free from any residual electron density suggesting that they are free from any solvent or water.

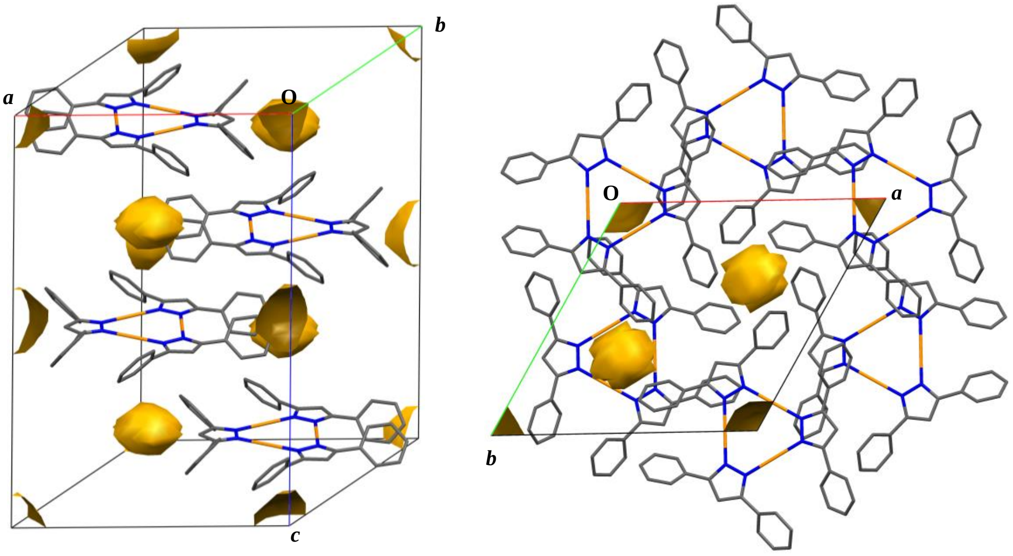

Figure 1. left) Unit cell contents of 4 displaying voids generated by Mercury[1], grid spacing = 0.7 probe radius = 1.2. Only molecules directly stacking above and below the void in the unit cell have been shown. Right) Only molecules with a phenyl ring in contact with the right void have been displayed.

Compound **3** possess larger voids of 426 Ǻ^3 making up 13.6 % the structure. The voids present within **3** differ from **4** as they are solvent accessible, running the entire length of the crystal, along the *a* axis, parallel to stacking of the gold (I) trimers as shown in Figure 2. The diameter of each

pore is approximately 7 nm, classifying the compound in the solid state as a mesoporous material, within the 2-50 nm range defined by IUPAC.[33]

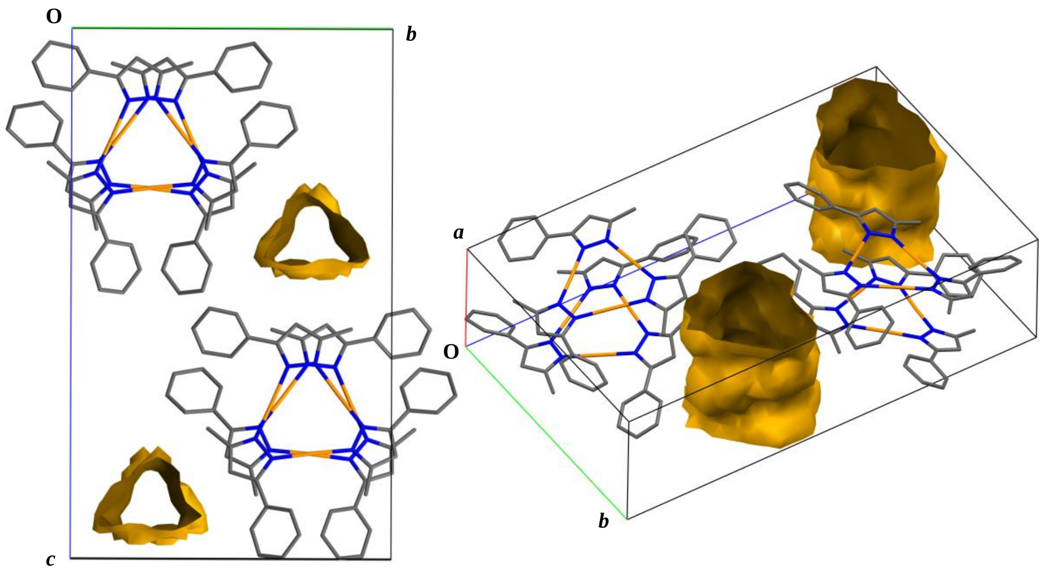

Figure 2. Unit cell contents of 3 with voids generated by mercury, grid space = 0.7, probe radius = 1.2

Due to larger solvent accessible voids in **3** it is not surprising that disordered solvent, THF, is present within the voids. TGA analysis using a Perkin Elmer TGA 4000 Thermogravimetric Analyzer. reveals that THF is present in approximately stoichiometric quantities with one mole of freshly crystallised **3** yielding one mole of THF released upon heating, the solvent dissociating from the material between a range of 150 to 170 degrees.

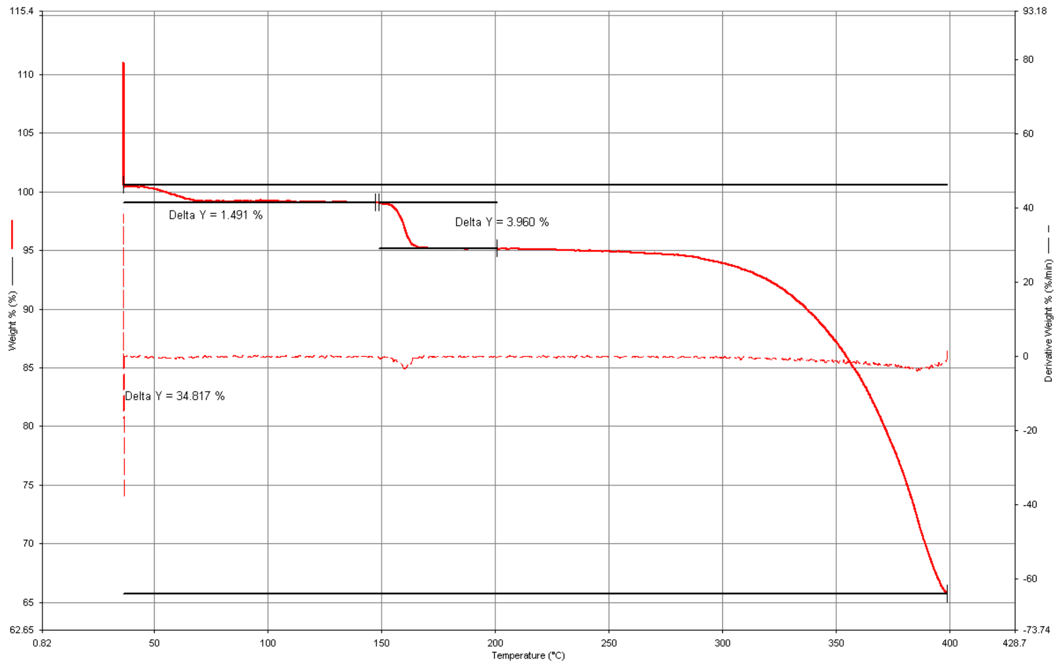

Figure 3. TGA analysis of 3

Void Analysis with temperature

Compound **3** and **4** possess significant voids in their structures. The voids present in both structures undergo faster contraction than the rest of the contents of the unit cell. At 293 K the void present in **4** occupies 33.2 % of the total unit cell volume calculated using Mercury (probe raduis = 0.2 ; grid spacing = 0.1), however at 100 K it now only occupies 30.5 %, a reduction of 2.7 %. Compound **3** undergoes similar behaviour reducing from 38.5 to 35.2 %, a reduction of 3.3 %.

Table 1. Void percentage of total unit cell volume for **2** and **3** change with temperature

K	Total unit cell volume (%)	
Temperature	**3**	**4**
100	35.3	30.5
120	35.5	30.9
150	35.9	31.2
180	36.3	31.5
210	36.8	32.0
240	37.3	32.4
270	37.9	32.9
293	38.5	33.2

In **3** there is no observed ordering of the solvent present upon cooling suggesting that the occupants of the void are ordered over several sites.

Void analysis with pressure

It was observed with temperature that the void present in the structure contracts significantly more than the rest of the structure and a similar phenomenon is observed with pressure, with voids contracting from 33.2 % to 21.4 % of the structure at 2.31 GPa in **4**.

Table 2. Percentage of total unit cell volume for **2** with compression.

**GPa**	**Total unit cell volume (%)**
Pressure	**4**
0.00†	33.1
0.52	28.9
0.93	26.0
1.24	24.9
1.31	24.6
1.76	22.9
2.05	22.0
2.31	21.4

† Ambient pressure values taken from 293 K structure of the variable temperature experiment

Compound **3** with its larger voids can also be analysed to a limited degree, observing a 3.90 % compression of void volume between 0.00 and 0.17 GPa with compression occurring in a similar manner to that observed with temperature

Relation of thermal indicatrix to unit cell contents

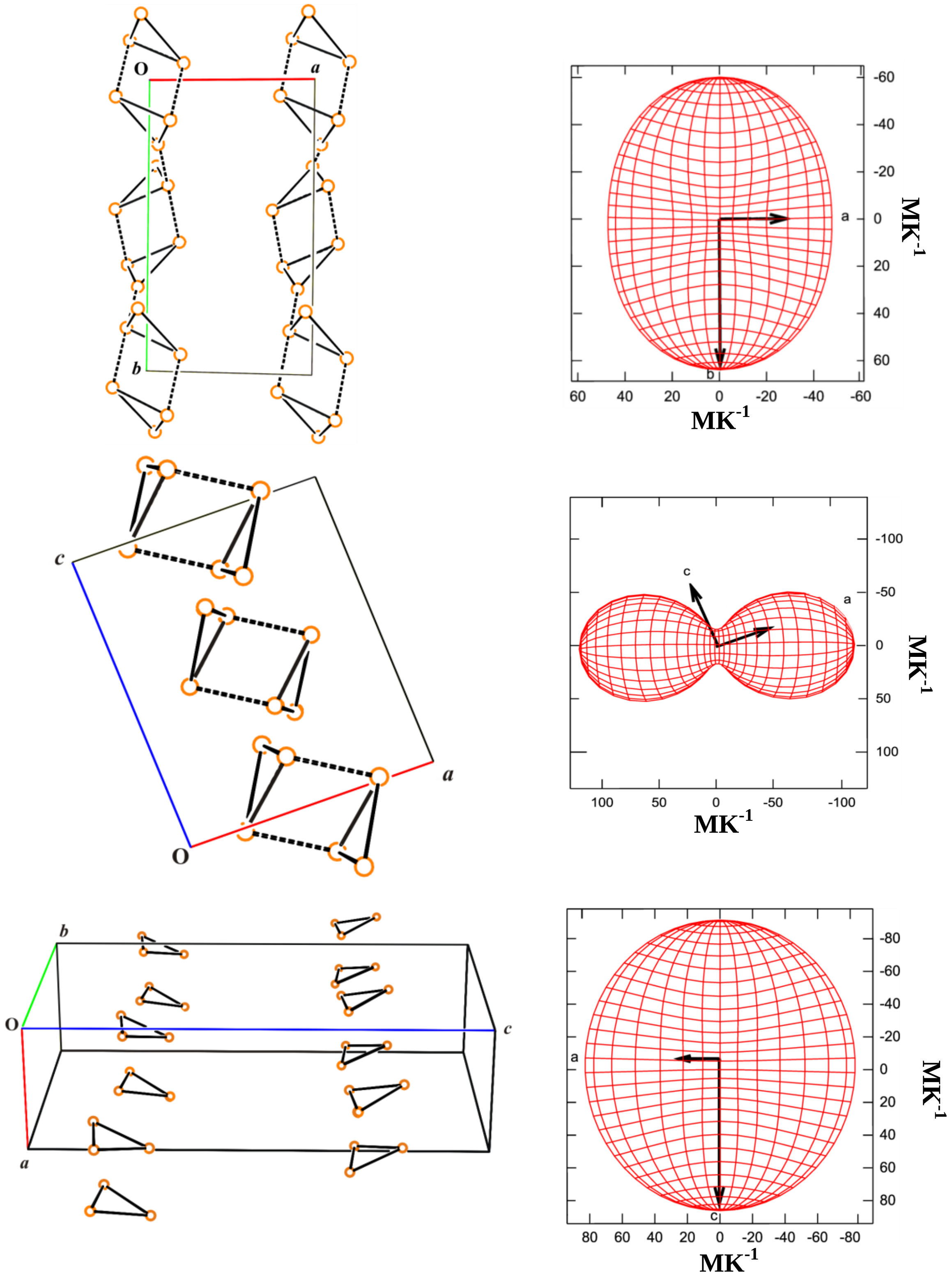

Figure 4. Top) Unit cell contents of 1 with only gold displayed and the thermal indicatrix upon contraction. Middle) Unit cell contents of **2** with only gold displayed and the thermal indicatrix upon contraction. Bottom) Unit cell contents of **3** with only gold displayed and the thermal indicatrix upon contraction.The indicatrix were calculated using PASCal.[2]

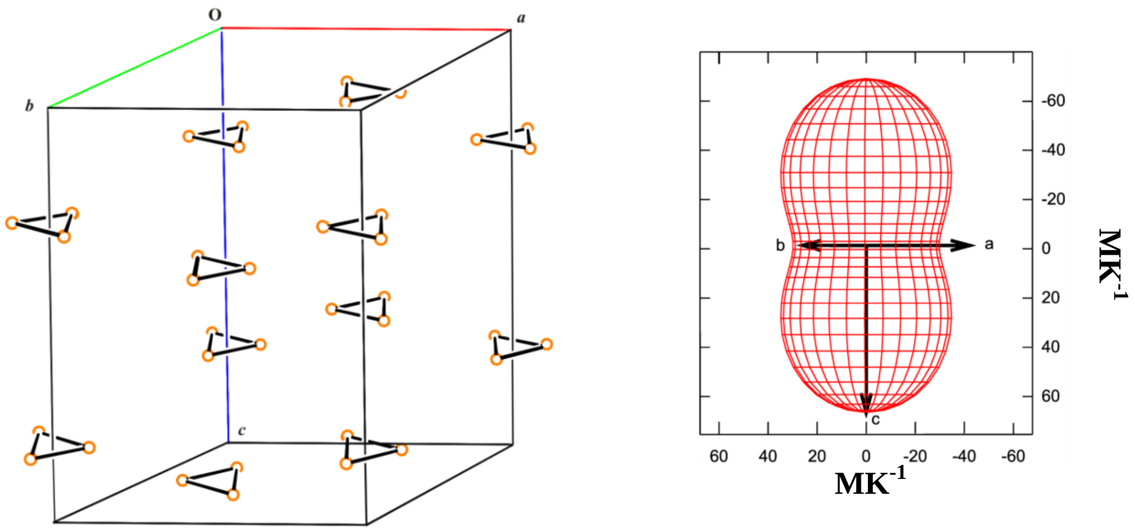

Figure 5. Unit cell contents of **4** with only gold displayed and the thermal indicatrix upon contraction

Equation of State Details

Table 3- Tris(μ_2-pyrazolato-N,N')-tri-gold(I) equations of state calculations from EOSFit[3]

**Equation fitted**	**Parameter**					
	V_0	K_0	K’	K’’	X^2w	$delP_{max}$
**Murnaghan**	1265.9(6)	10.3(5)	6.8(3)	-	3.5733	0.123
**Birch Murnaghan 2nd order**	1265.5(2.4)	15.7(6)	4	-	66.6980	-0.567
**Birch Murnaghan 3rd order***	1265.9(4)	8.69(48)	10.69(81)	-6.38	1.716	0.099
**Birch Murnaghan 4th order**	1265.9(3)	6.27(1.1)	20.98(6.5)	-57.89	0.9060	0.072

Table 4 - Tris(μ_2-3,4,5-trimethylpyrazolato-N,N')-tri-gold(I) equations of state calculations from EOSFit

**Equation fitted**	**Parameter**					
	V_0	K_0	K’	K’’	X^2w	$delP_{max}$
**Murnaghan**	2168.2(1)	7.7(6)	7.2(5)		0.2296	-0.098
**Birch Murnaghan 2nd order**	2168.9(3)	12.7(4)	4		9.8898	-0.461
**Birch Murnaghan 3rd order***	2168.3(1)	6.3(8)	12.0(2.07)	-11.80	0.1450	-0.109
**Birch Murnaghan 4th order**	2168..3(1)	6.1(2.7))	13.2(12.5)	-16.88(57)	0.1787	-0.114

Table 5 - Tris(μ_2-3,5-diphenylpyrazolato-N,N')-tri-gold(I) contraction from 0.0001 – 2.31 GPa

Equation fitted	Parameter					
	V_0	K_0	K'	K''	X^2w	delP$_{max}$
**Murnaghan**	5748.4(2)	6.5(7)	10.3(1.3)	-	0.1324	0.046
**Birch Murnaghan 2nd order**	5748.5(4)	10.5(4)	4	-	4.1655	0.229
**Birch Murnaghan 3rd order***	5748.5(2)	5.42(1.05)	18.35(5.6)	-41.35	0.1643	-0.040
**Birch Murnaghan 4th order**	5748.5(2)	7.33(4.65)	5.35(23.00)	14.977()30.5	0.1612	0.050

Calculated axes of compression and contraction

Table 6 - Tris(μ_2-pyrazolato-N,N')-tri-gold(I) (1) axes of contraction from 293 K – 100 K

Axes of contraction			Direction		
	$\alpha(MK^{-1})$	$\sigma\ \alpha\ (MK^{-1})$	a	b	c
**X1**	24.1253	0.5904	0.5042	0.000	-0.8636
**X2**	47.6110	0.5311	0.5042	0.000	0.4390
**X3**	62.0038	1.5331	0.000	-1.0000	0.000
**V**	135.2234	2.6619			

Table 7 - Tris(μ_2-pyrazolato-N,N')-tri-gold(I) (1) axes of compression from 0.0001 –7.80 GPa

Axes of compression			Direction		
	$K(TPa^{-1})$	$\sigma\ K\ (TPa^{-1})$	a	b	c
**X1**	10.2331	0.4830	0.000	1.0000	0.000
**X2**	6.0240	0.2060	0.9712	0.000	0.2381
**X3**	4.2661	0.2117	-0.0505	0.000	0.9987
**V**	30.5132	4.5792			

Table 8 - Tris(μ_2-3,4,5-trimethylpyrazolato-N,N')-tri-gold(I) axes of contraction from 293 K – 100 K

Axes of contraction			Direction		
	$\alpha(MK^{-1})$	$\sigma\ \alpha\ (MK^{-1})$	a	b	c
**X1**	6.3961	0.6081	-0.4295	0.000	-0.9031
**X2**	36.2658	0.3505	0.000	1.0000	0.000
**X3**	120.8530	1.4049	-0.9659	0.000	0.2588
**V**	164.9198	1.3254			

Table 9 - Tris(μ_2-3,4,5-trimethylpyrazolato-N,N')-tri-gold(I) axes of compression from 0.0001 – 5.18 GPa

Axes of compression			Direction		
	K(TPa^{-1})	σ K (TPa^{-1})	a	b	c
**X1**	18.8546	0.3309	-0.6612	0.000	0.7502
**X2**	8.5954	1.9230	0.8443	0.000	0.5358
**X3**	4.3897	0.3316	0.000	-1.000	0.000
**V**	42.3481	6.4114			

Table 10 - Tris(μ_2-3-phenyl-5-methylpyrazolato-N,N')-tri-gold(I) axes of contraction from 293 K – 100 K

Axes of contraction			Direction		
	α(MK^{-1})	σ α (MK^{-1})	a	b	c
**X1**	44.4475	0.1546	0.000	1.000	0.000
**X2**	80.6303	1.0409	-1.000	0.000	0.000
**X3**	84.7880	2.4335	0.000	0.000	1.000
**V**	213.0343	3.6941			

Table 11 - Tris(μ_2-3,5-diphenylpyrazolato-N,N')-tri-gold(I) contraction from 293 K – 100 K

Axes of contraction			Direction		
	α(MK^{-1})	σ α (MK^{-1})	a	b	c
**X1**	26.1152	3.2068	0.7071	-0.7071	0.000
**X2**	26.1152	3.2068	-0.7071	-0.7071	0.000
**X3**	67.7525	1.9167	0.000	0.000	1.000
**V**	122.7924	7.2546			

Table 12 - Tris(μ_2-3,5-diphenylpyrazolato-N,N')-tri-gold(I) contraction from 0.0001 – 2.31 GPa

Axes of compression			Direction		
	K(TPa^{-1})	σ K (TPa^{-1})	a	b	c
**X1**	23.3872	0.9906	0.000	0.000	1.000
**X2**	13.1963	4.4782	-0.0861	0.9963	0.000
**X3**	13.1965	4.4772	0.8717	0.4900	0.000
**V**	60.1796	6.7421			

Unusual crystallographic behaviour of compound 3

Data for **3** could only be collected up to a pressure of 0.17 GPa. Any attempt to increase the pressure higher resulted in a catastrophic change in the diffraction pattern that rendered the data unsolvable. With high pressure data it is common that a crystal is destroyed during the process of pressurisation. Crystal failure produces a diffraction pattern where the peaks are split, broad and smeary often in multiple domains and diffract only to a low theta angle. Above the pressure of 0.17 GPa the sample does not display any of these characteristics with only a slight reduction in resolution as shown in Figure 6.

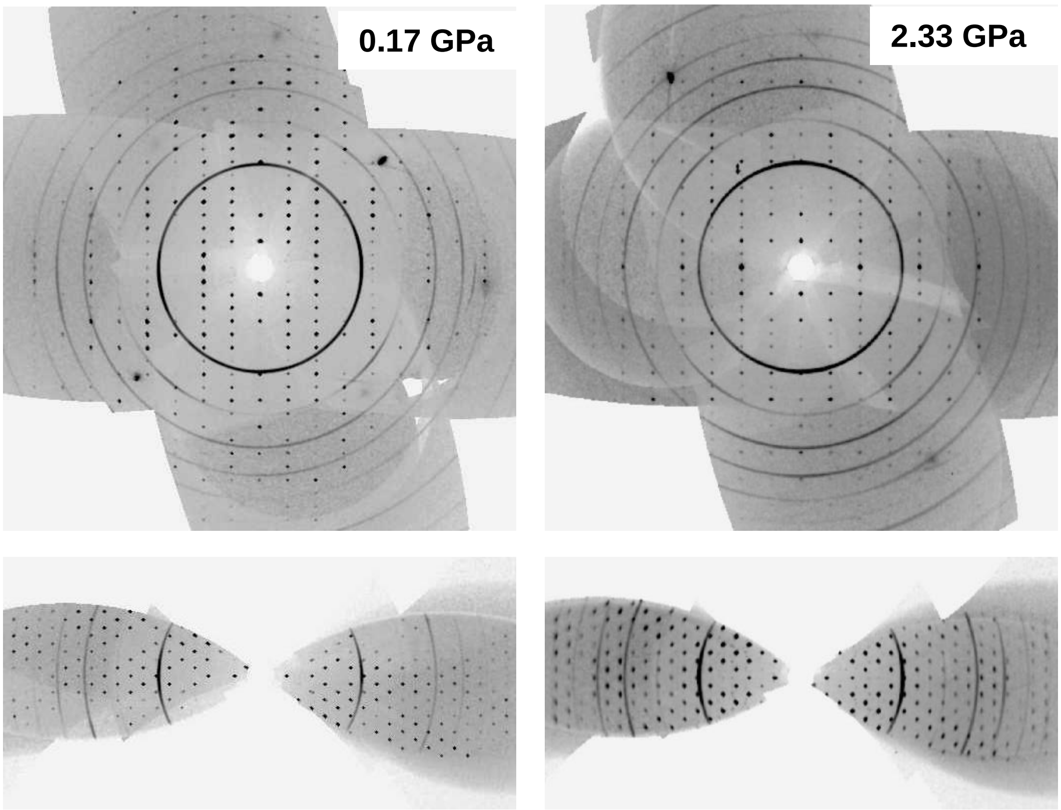

Figure 6 Top) Axial images of the (*hk0*) plane at 0,17 and 2.33 GPa Bottom) Axial images of (*0kl*) plane at 0.17 GPa and 2.33 GPa

Attempts at indexing the crystal reveals that the crystal undergoes a enormous change down the *l* axis as shown below in Figure 7. It is obvious from the images that no useful information is likely to come from the pattern as it is impossible to distinguish individual peaks from one another.

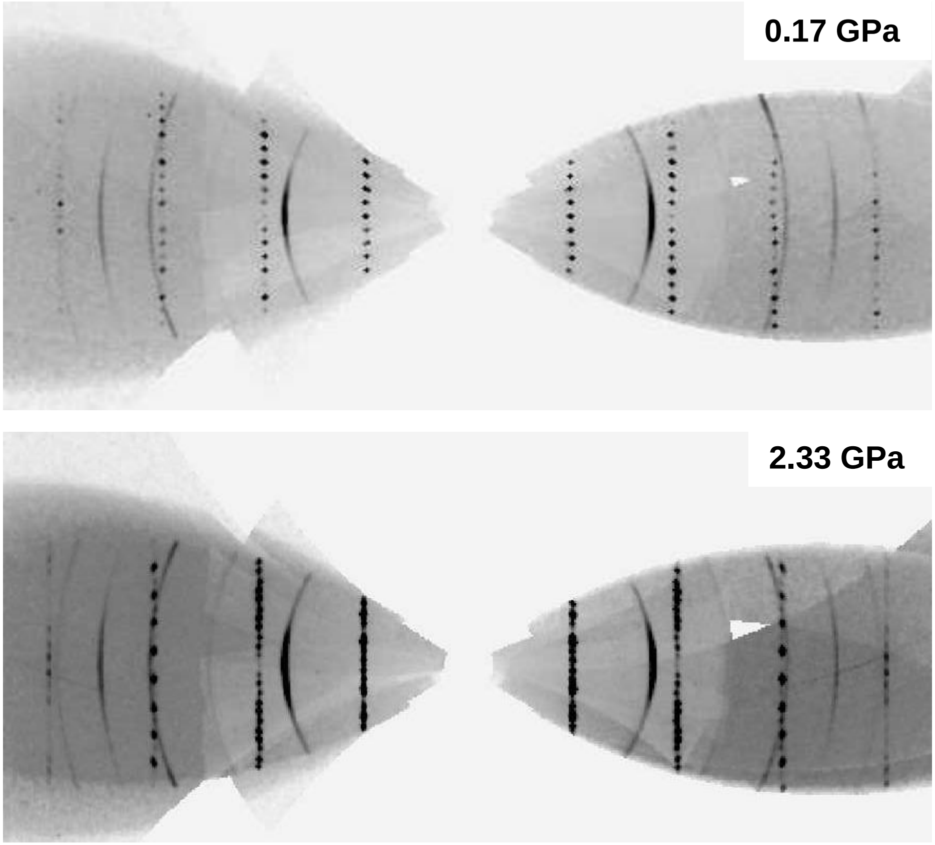

Figure 7. Axial image of (*h0l*) plane at 0.17 GPa and 2.33 GPa

This result is highly intriguing and difficult to explain without further information. Attempts were made to observe more data along the *l* axis but this proved impossible due to the crystal morphology restricting the manner in which the crystal may be loaded in the DAC.

Unusual crystallographic behaviour of compound 4

Excellent quality data was collected for **4** up to the pressure of 2.31 GPa and the data up to that point has formed the basis for analysis of **4** in the previous sections. Upon further compression it was noted that the crystal no longer produced bragg peaks upon exposure to X-rays, suggesting that the sample had become amorphous or non-crystalline.

Further compression up to 3.66 GPa saw the re-introduction of bragg peaks to the diffraction pattern although they were of very poor quality, smeared, streaky and not all of one clear domain. From the peaks a new unit cell was found of a *C2/c* cell. Comparison of the new cell to the previous *R-3c* converted to a monoclinic cell suggests the possible occurrence of a first order phase transition, with a discontinuity in volume shown in Figure 8.

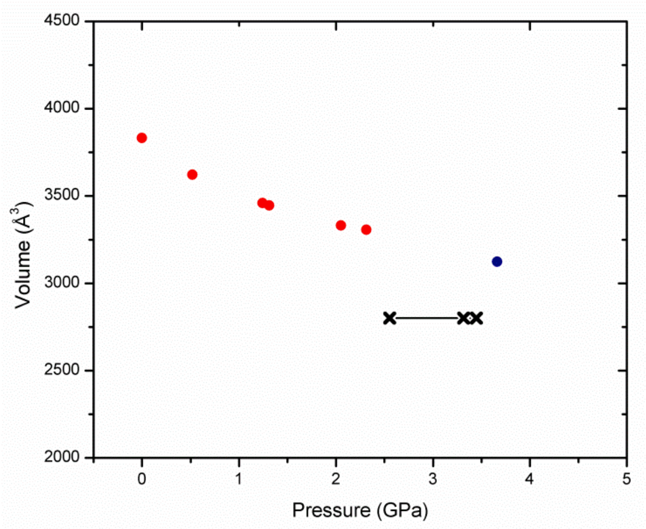

Figure 8. Unit cell volume of **4**, the know *R-3c* cell in red having been converted to a monoclinic cell. The new possible phase shown in blue. Crosses indicate the pressures taken where no diffraction was observed from the sample.

An important point to mention is that upon relaxation of pressure, a previously diffractionless crystal in the region above 2.31 GPa regained strong diffraction again at lower pressure in the original R-3c cell setting as shown in Figure 9 suggesting that the process occurring is reversible and non-destructive to the crystal.

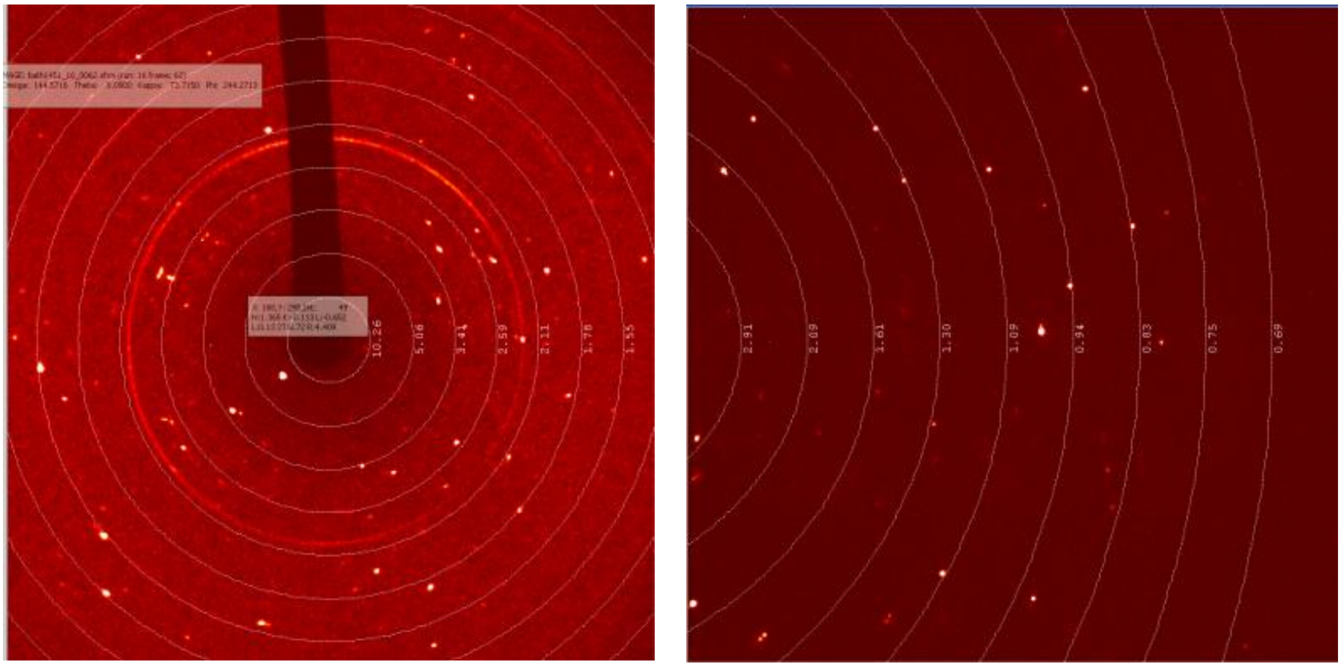

Figure 9. Left) Diffraction of 4 at 3.66 GPa. Right) Diffraction of 4 at 0.00 GPa after being at 3.66 GPa

Behaviour of this sort is clearly unusual and intriguing but unfortunately without further study it is difficult to draw any conclusion about what the processes may be involved. It should be noted that while such behaviour is unique in the literature with respect to pressure there are several examples of similar behaviour regarding MOFs and zeolites which have been shown to display similar properties when solvating and desolvating at high temperature.[4] It is possible that similar behaviour could be being observed here with pressure forcing hydrostatic media into the voids present within the structure, although this is conjecture.

Analysis of non-aurophilic interactions with pressure

Pressure is an effective tool for altering other interactions within a crystal structure. Hirshfeld surface analysis of the data at varying pressure demonstrates how some of the interactions become more pronounced as the pressure increases and provide further evidence that there are no dramatic changes in crystal packing motifs.

The C-H…π interactions present in the ambient structure of **1** become more prevalent in the plot, indicated in Figure 10 at elevated pressure indicating that the distance of the interaction reducing more than other interactions in the structure.

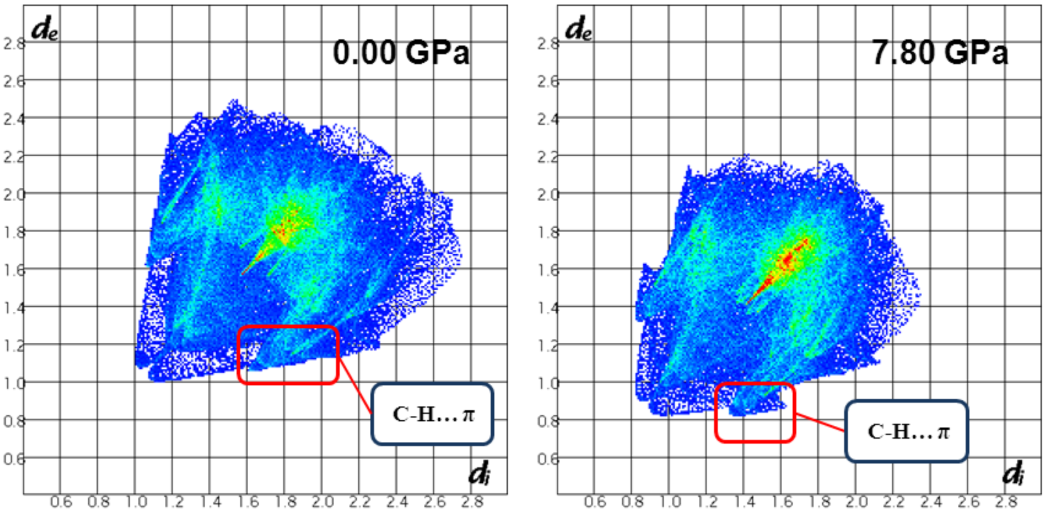

Figure 10. Fingerprint plot of 1 at 0.00 and 7.80 GPa respectively

Plots of **2** reveal an increase in C-H…H steric interactions with the appearance of protrusion along the diagonal, highlighted in the bottom pictures of The fingerprint plot shows how with compression the molecules of **2** are forced together in the direction of the trimer stacking despite increasing destabilising steric interactions. It has been suggested a $D_i + D_e$ value of 1.7 Å in a fingerprint plot represents an absolute limit of H…H interaction below which it is not possible to compress such interactions.[5] It is clear from the 5.31 GPa fingerprint plot of Figure 11 that this value has been surpassed in the case of **2** with a $D_i + D_e$ of approximately 1.6 Å.

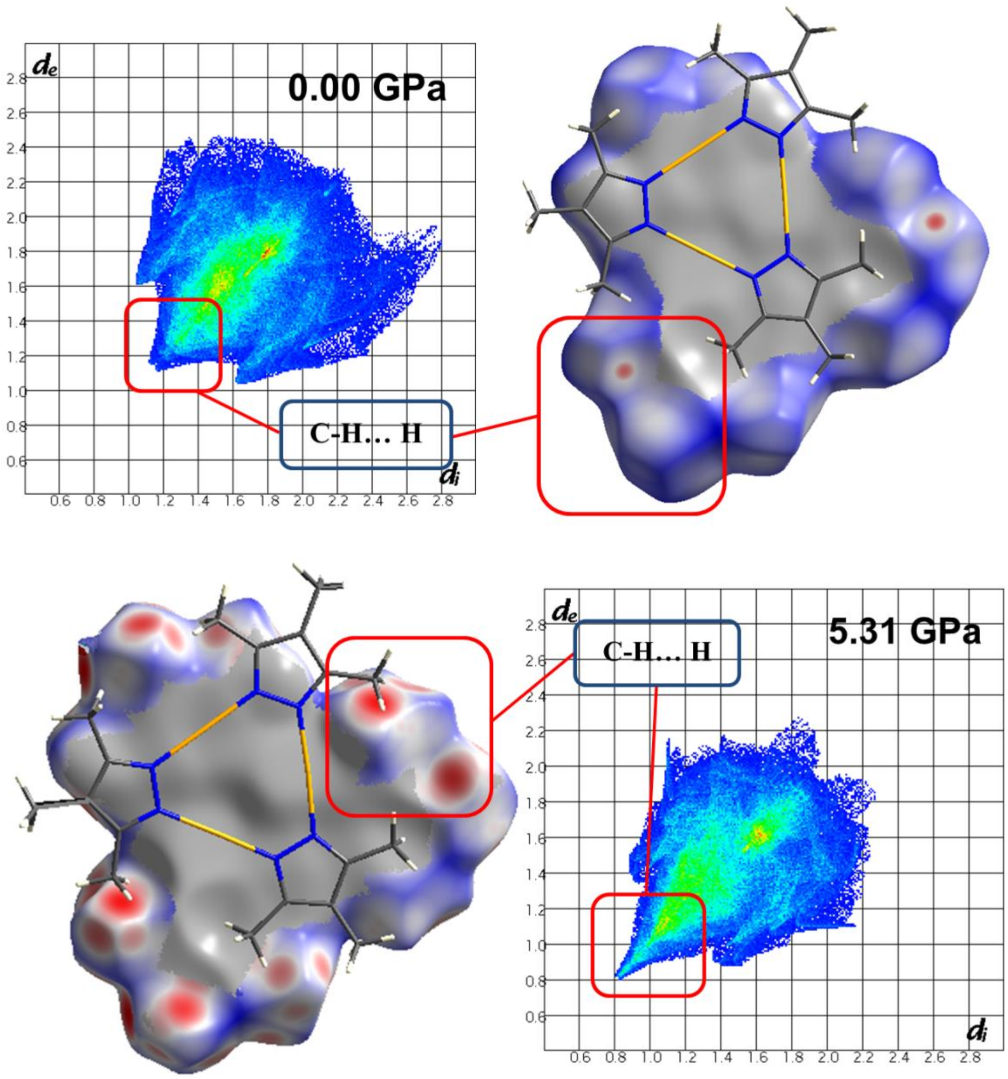

Figure 11. Top) D_{norm} Hirshfeld surface of **4** at 0.00 GPa with respective fingerprint plot. Bottom) D_{norm} hirschfeld surface of **4** at 5.31 GPa with respective fingerprint plot. Both surfaces display only the interactions involved in C-H…H interactions

Raman Spectroscopy

Raman spectroscopy compliments the luminescence spectra well. The spectra of **1** display some subtle but significant changes throughout the temperature range studied indicating a structural change in the complex with change in temperature.

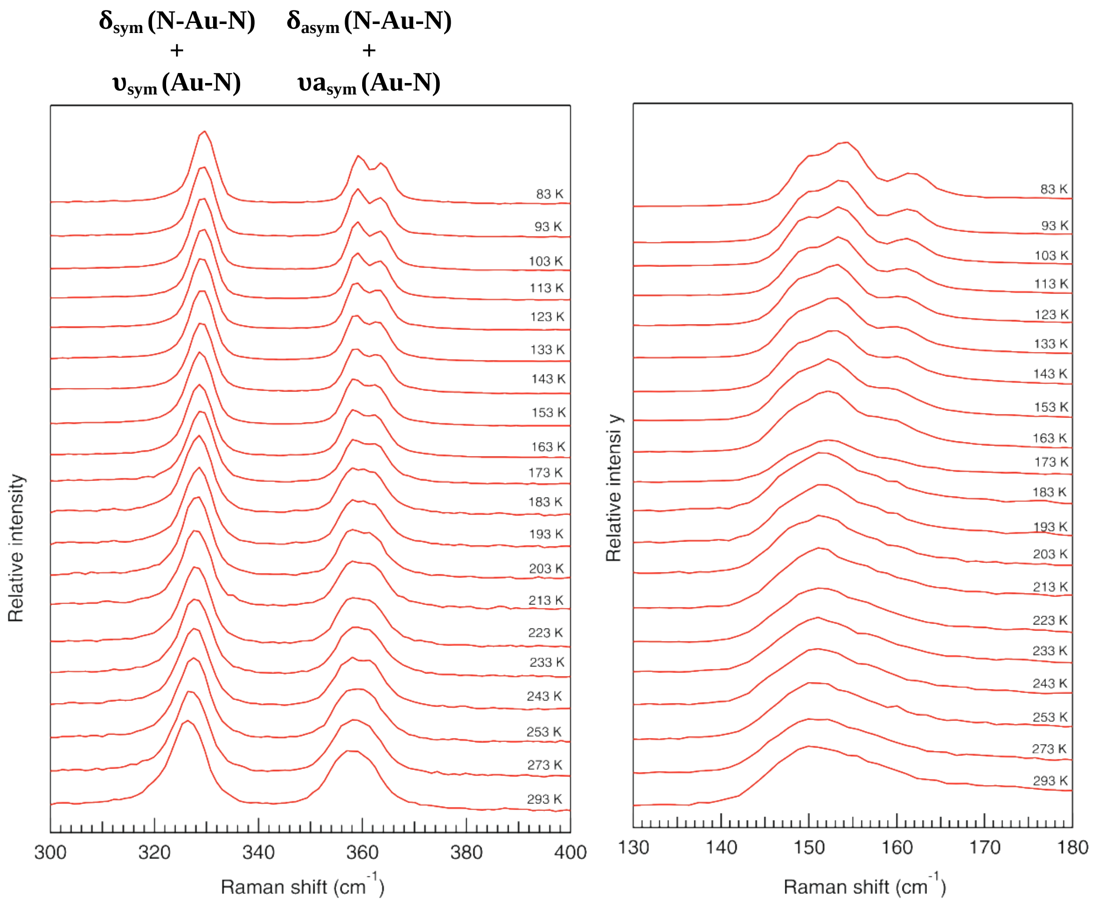

Figure 12. Left) Variable temperature raman spectroscopy of **1** of the 300-400 cm^{-1}region. Right) and the 130-180 cm^{-1} region.

Variable temperature Raman demonstrates the expected redshift in the symmetric and asymmetric N-Au-N stretches and bends with increasing temperature consistent with a slight weakening of the bonding. A similar trend is also observed in regions of the spectra associated with Au….Au interactions.

Computational band gap changes.

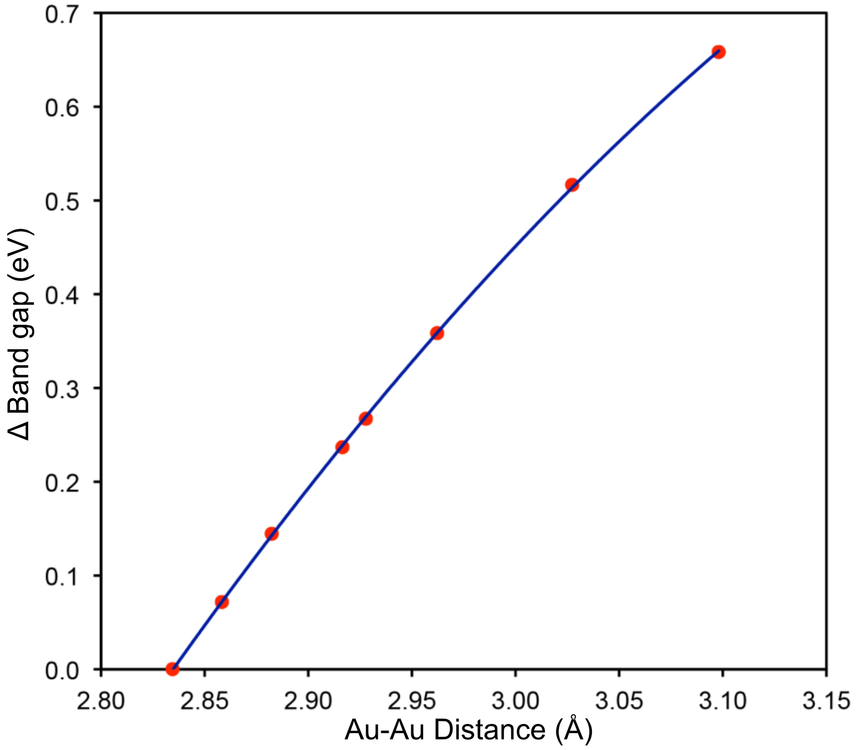

Figure 13 - Calculated change in band gap for 1 as a function of Au-Au distance, as determined from the pressure-dependence of the crystal and electronic structure.

Crystallography tables

Table 13. Crystallographic data of tris(μ_2-Pyrazolato-N,N')-tri-gold(I) at variable temperature

**Temperature(K)**	**293**	**270**	**240**	**210**	**180**	**150**	**120**	**100**
**Formula**	$C_9H_9N_2Au_3$	$C_9H_9N_2Au_3$	$C_9H_9N_2Au_3$	$C_9H_9N_2Au_3$	$C_9H_9N_2Au_3$	$C_9H_9N_2Au_3$	$C_9H_9N_2Au_3$	$C_9H_9N_2Au_3$
**M_r**	792.12	792.12	792.12	792.12	792.12	792.12	792.12	792.12
**Crystal System**	Monoclinic	Monoclinic	Monoclinic	Monoclinic	Monoclinic	Monoclinic	Monoclinic	Monoclinic
**Space Group**	$P2_1/c$	$P2_1/c$	$P2_1/c$	$P2_1/c$	$P2_1/c$	$P2_1/c$	$P2_1/c$	$P2_1/c$
**a,b,c (Å)**	8.3340(12)<br>14.364(2)<br>11.0508(16)	8.3262(12)<br>14.342(2)<br>11.0439(15)	8.3155(10)<br>14.3123(18)<br>11.0338(13)	8.3052(9)<br>14.2829(16)<br>11.0235(12)	8.2962(10)<br>14.2537(17)<br>11.0138(13)	8.2870(11)<br>14.2316(18)<br>11.0070(14)	8.2776(11)<br>14.2092(18)<br>10.9986(14)	8.2722(17)<br>14.196(3)<br>10.994(2)
**β (°)**	106.890(2)	106.907(2)	106.9310(10)	106.9710(10)	107.0050(10)	107.045(2)	107.075(2)	107.09(3)
**V ($Å^3$)**	1265.9(3)	1261.8(3)	1256.3(3)	1250.7(2)	1245.5(3)	1241.1(3)	1236.6(3)	1234.1(4)
**Z/Z'**	4/1	4/1	4/1	4/1	4/1	4/1	4/1	4/1
**D_x (Mg m^{-3})**	4.156	4.170	4.188	4.207	4.224	4.239	4.255	4.264
**μ(mm^{-1})**	34.673	42.066	42.252	42.440	42.618	42.767	42.923	43.012
**F(000)**	1368	1368	1368	1368	1368	1368	1368	1368
**T_{min} /T_{max}**	0.38870.7468	0.4180/0.7468	0.4522/0.7468	0.4509/0.7469	0.4630/0.7469	0.4584/0.7468	0.4681/0.7469	0.4719/0.7468
**2θ range(°)**	2.7\8/34.68	2.79/34.70	2.79/34.74	2.80/34.78	2.80/34.82	3.12/ 34.70	2.81/ 34.78	2.43/29.55
**Rint**	0.0856	0.0812	0.0753	0.0686	0.0691	0.0682	0.0660	0.0632
**No. Measured/ Independent Observed reflections**	13723/4012	13546/3973	13520/3980	134573968	13257/3949	13136/ 3933	13065/ 3918	13001/ 3890
**R[F]/wR[F2] (for I >2σ)**	0.0484/0.1347	0.0435/0.1025	0.0417/0.1039	0.0383/0.0949	0.0411/0.1072	0.0398/ 0.1062	0.0409/ 0.1133	0.0375/ 0.1010
**R[F]/wR[F2] (all data)**	0.0613/0.1491	0.0550/0.1104	0.0506/0.1101	0.0459/0.0999	0.0467/ 0.1107	0.0450/ 0.1095	0.0452/ 0.1160	0.0413/ 0.1038
**Restraints/Parameters**	0/164	0/164	0/163	0/164	0/164	0/164	0/164	0/ 164
**$\Delta\rho_{max}$,$\Delta\rho_{min}$ (e $Å^{-3}$)**	1.942/-2.170	1.806/-1.848	1.967/-1.727	1.580/-1.786	1.900/-2.412	1.980/-3.231	2.628/.-2.331	2.363/-2174
**REFCODE**	971102	971101	971100	971099	971098	971097	971096	971095

Table 14 Crystallographic data of tris(μ_2-3,4,5-trimethylpyrazolato-N,N')-tri-gold(I) at variable temperature

Temperature(K)	293	270	240	210	180	150	120	100
**Formula**	$C_{18}H_{27}N_2Au_3$	$C_{18}H_{27}N_2Au_3$	$C_{18}H_{27}N_2Au_3$	$C_{18}H_{27}N_2Au_3$	$C_{18}H_{27}N_2Au_3$	$C_{18}H_{27}N_2Au_3$	$C_{18}H_{27}N_2Au_3$	$C_{18}H_{27}N_2Au_3$
**M_r**	918.36	918.36	918.36	918.36	918.36	918.36	918.36	918.36
**rystal System**	Monoclinic	Monoclinic	Monoclinic	Monoclinic	Monoclinic	Monoclinic	Monoclinic	Monoclinic
**Space Group**	$P2_1/c$	$P2_1/c$	$P2_1/c$	$P2_1/c$	$P2_1/c$	$P2_1/c$	$P2_1/c$	$P2_1/c$
**a,b,c (Å)**	8.9753(2)<br>22.3828(6)<br>10.8274(3)	8.9485(2)<br>22.3601(6)<br>10.8249(3)	8.9143(2)<br>22.3356(5)<br>10.8211(3)	8.8835(2)<br>22.3125(5)<br>10.8161(3)	8.8557(2)<br>22.2874(5)<br>10.8097(3)	8.8277(2)<br>22.2643(5)<br>10.8012(3)	8.8004(2)<br>22.2401(5)<br>10.7923(2)	8.7843(2)<br>22.2261(6)<br>10.7865(2)
**β (°)**	94.550(3)	94.478(3)	94.370(3)	94.256(3)	94.137(2)	94.016(2)	93.908(2)	93.822(2)
**V (Å^3)**	2168.29(10)	2159.34(10)	2148.28(9)	2137.98(9)	2127.96(9)	2117.68(9)	2107.38(8)	2101.28(8)
**Z/Z'**	4/1	4/1	4/1	4/1	4/1	4/1	4/1	4/1
**D_x (Mg m^{-3})**	2.813	2.825	2.839	2.853	2.867	2.880	2.895	2.903
**μ(mm^{-1})**	20.263	20.347	20.452	20.551	20.647	20.747	20.849	20.909
**F(000)**	1656	1656	1656	1656	1656	1656	1656	1656
**T_{min} /T_{max}**	0.70327/1.0000	0.71845/1.0000	0.72273/1.0000	0.7181/1.0000	0.716431.000/	0.70702/1.0000	0.70646/1.0000	0.70512/1.0000
**2θ range(°)**	2.91/26.37	2.99/29.13	3.00/32.87	3.01/32.86	3.02/32.89	3.02/32.89	3.03/32.93	3.04/32.95
**Rint**	0.0474	0.0652	0.0694	0.0652	0.0641	0.0598	0.0583	0.0576
**No. Measured/ Independent Observed reflections**	19262/4420	15439/5653	17752/7074	17698/7030	17493/6993	17436/6961	17036/6908	17203/6913
**R[F]/wR[F2] (for I >2σ)**<br>**R[F]/wR[F2] (all data)**	0.0320/0.0554<br>0.0561/0.0609	0.0453/0.0631<br>0.0930/0.0770	0.0501/0.0682<br>0.1182/0.0887	0.0491/0.0680<br>0.1047/0.0835	0.0473/0.0666<br>0.0975/0.0820	0.0454/0.0708<br>0.0805/0.0824	0.0442/0.0708<br>0.0776/0.0811	0.0432/0.0675<br>0.0748/0.0768
**Restraints/Parameters**	0/253	0/253	0/253	0/253	0/253	0/253	0/253	0/253
**$\Delta\rho_{max}$,$\Delta\rho_{min}$ (e Å^{-3})**	1.359/-0.893	1.732/-1.628	2.033/-1.733	1.803/-2.466	1.878/-1.908	2.486/-1.932	2.371/-1.731	2.497/-2.187
**REFCODE**	971128	971126	971124	9711213	971122	971121	971120	971119

Table 15 Crystallographic data of tris(μ_2-3-methyl-5-phenylpyrazolato-N,N')-tri-gold(I) at variable temperature

**Temperature(K)**	**293**	**270**	**240**	**210**	**180**	**150**	**120**	**100**
**Formula**	$C_{30}H_{27}N_6Au_3$	$C_{30}H_{27}N_6Au_3$	$C_{30}H_{27}N_6Au_3$	$C_{30}H_{27}N_6Au_3$	$C_{30}H_{27}N_6Au_3$	$C_{30}H_{27}N_6Au_3$	$C_{30}H_{27}N_6Au_3$	$C_{30}H_{27}N_6Au_3$
**M_r**	1062.48	1062.48	1062.48	1062.48	1062.48	1062.48	1062.48	1062.48
**Crystal System**	orthorhombic	orthorhombic	orthorhombic	orthorhombic	orthorhombic	orthorhombic	orthorhombic	orthorhombic
**Space Group**	$P\ n\ a\ 2_1$	$P\ n\ a\ 2_1$	$P\ n\ a\ 2_1$	$P\ n\ a\ 2_1$	$P\ n\ a\ 2_1$	$P\ n\ a\ 2_1$	$P\ n\ a\ 2_1$	$P\ n\ a\ 2_1$
**a,b,c (Å)**	7.4065(10)<br>16.001(2)<br>26.502(3)	7.3869(7)<br>15.9840(15)<br>26.454(2)	7.3652(7)<br>15.9628(15)<br>26.388(2)	7.3454(8)<br>15.9427(18)<br>26.326(3)	7.3263(6)<br>15.9195(13)<br>26.255(2)	7.3104(6)<br>15.8993(12)<br>26.205(2)	7.2939(6)<br>15.8792(13)<br>26.144(2)	7.2843(6)<br>15.8645(12)<br>26.101(2)
**β (°)**	90	90	90	90	90	90	90	90
**V ($Å^3$)**	3140.9(7)	3123.5(5)	3102.4(5)	3082.9(6)	3062.1(4)	3045.8(4)	3028.1(4)	3016.2(4)
**Z/Z'**	4/1	4/1	4/1	4/1	4/1	4/1	4/1	4/1
**D_x (Mg m^{-3})**	2.247	2.259	2.275	2.289	2.305	2.305	2.331	2.340
**μ(mm^{-1})**	16.923	17.018	17.134	17.242	17.359	17.359	17.554	17.623
**F(000)**	1944	1944	1944	1944	1944	1944	1944	1944
**T_{min} /T_{max}**	0.5809/0.7469	0.5998/0.7469	0.5998/0.7466	0.5879/0.7465	0.5789/0.7355	0.5672/0.7469	0.5775/0.7368	0.5847/0.7225
**2θ range(°)**	2.80/29.64	3.25/34.87	3.25/33.51	2.89/33.03	2.90/34.45	2.90/34.83	2.91/34.82	2.57/31.61
**Rint**	0.0587	0.0586	0.0581	0.0676	0.0688	0.0667	0.0678	0.0772
**No. Measured/ Independent Observed reflections**	43207/10119	42847/10072	30788/9008	30007/8691	31156/9367	31077/9349	30676/9284	31427/9599
**R[F]/wR[F2] (for I >2σ)**<br>**R[F]/wR[F2] (all data)**	0.0466/0.1177<br>0.0559/0.1224	0.0307/0.0709<br>0.0360/0.0734	0.0311/0.0709<br>0.0347/0.0729	0.0489/0.1456<br>0.0526/0.1497	0.0359/0.0892<br>0.0394/0.0911	0.0468/0.1219<br>0.0501/0.1236	0.0493/0.1306<br>0.0524/0.1326	0.0379/0.0909<br>0.0415/0.0933
**Restraints/Parameters**	1/355	1/355	1/355	1/355	1/355	1/355	1/355	1/355
**$\Delta\rho_{max}$,$\Delta\rho_{min}$ (e $Å^{-3}$)**	1.075/-0.878	0.813/-0.886	0.685/-0.577	1.651/-1.211	1.300/-1.214	2.074/-1.118	2.319/-1.510	1.327-1.051
**REFCODE**	971111	971112	971113	971114	971115	971116	971117	971118

Table 16. Crystallographic data of tris(μ_2-3,5-diphenylpyrazolato-N,N')-tri-gold(I) at variable temperature

**Temperature(K)**	**293**	**270**	**240**	**210**	**180**	**150**	**120**	**100**
**Formula**	$C_{45}H_{33}N_6Au_3$	$C_{45}H_{33}N_6Au_3$	$C_{45}H_{33}N_6Au_3$	$C_{45}H_{33}N_6Au_3$	$C_{45}H_{33}N_6Au_3$	$C_{45}H_{33}N_6Au_3$	$C_{45}H_{33}N_6Au_3$	$C_{45}H_{33}N_6Au_3$
**M_r**	1248.67	1248.67	1248.67	1248.67	1248.67	1248.67	1248.67	1248.67
**Crystal System**	Trigonal	Trigonal	Trigonal	Trigonal	Trigonal	Trigonal	Trigonal	Trigonal
**Space Group**	*R-3c*	*R-3c*	*R-3c*	*R-3c*	*R-3c*	*R-3c*	*R-3c*	*R-3c*
**a,b,c (Å)**	16.0783(3)<br>16.0783(3)<br>25.6914(7)	16.05410(10)<br>16.05410(10)<br>25.6344(3)	16.0514(2)<br>16.0514(2)<br>25.5870(3)	16.0355(2)<br>16.0355(2)<br>25.5302(3)	16.0222(2)<br>16.0222(2)<br>25.4804(3)	16.00990(10)<br>16.00990(10)<br>25.4435(3)	15.9968(2)<br>15.9968(2)<br>25.3942(3)	15.97110(10)<br>15.97110(10)<br>25.3408(3)
**γ(°)**	120	120	120	120	120	120	120	120
**V ($Å^3$)**	5751.7(2)	5721.70(8)	5709.20(12)	5685.25(12)	5664.75(12)	5647.87(8)	5627.70(12)	5597.84(8)
**Z/Z'**	6/1/3	6/1/3	6/1/3	6/1/3	6/1/3	6/1/3	6/1/3	6/1/3
**D_x (Mg m^{-3})**	2.163	2.174	2.179	2.188	2.196	2.203	2.211	2.222
**μ(mm^{-1})**	11.491	11.551	11.577	11.625	11.667	11.702	11.744	11.807
**F(000)**	3492	3492	3492	3492	3492	3492	3492	3492
**T_{min} /T_{max}**	0.69800/1.000	0.44011/1.000	0.45247/1.000	0.45999/1.000	0.45480/1.000	0.48461/1.000	0.46825/1.000	0.48154/1.000
**2θ range(°)**	3.33/32.84	4.19/32.90	4.19/ 32.86	4.20/32.76	4.20/32.81	4.21/32.84	4.21/32.76	4.22/32.83
**Rint**	0.0367	0.0375	0.0368	0.0360	0.0362	0.0356	0.0349	0.0346
**No. Measured/ Independent Observed reflections**	22942/2283	45404/2304	45677/2299	45105/2277	45086/2271	45654/2264	44828/2254	44498/2242
**R[F]/wR[F2] (for I >2σ)**<br>**R[F]/wR[F2] (all data)**	0.0264/0.0502<br>0.0395/0.0543	0.0186/0.0402<br>0.0250/0.0425	0.0177/0.0404<br>0.0221/0.0419	0.0171/0.0377<br>0.0210/0.0387	0.0166/0.0374<br>0.0195/0.0381	0.0157/0.0388<br>0.0180/0.0396	0.0149/0.0371<br>0.0167/0.0376	0.0151/0.0389<br>0.0164/0.0392
**Restraints/Parameters**	0/40	0/83	0/83	0/83	0/83	0/83	0/83	0/83
**$\Delta\rho_{max}$,$\Delta\rho_{min}$ (e $Å^{-3}$)**	0.881/-0.924	0.540/-1.155	0.576/-1.201	0.540/-1.240	0.510/-1.317	0.603/-1.378	0.574/-1.352	0.612/-1.328
**REFCODE**	971110	971109	971108	971107	971106	971105	971104	971103

Table 17. Crystallographic data of tris(μ_2-Pyrazolato-N,N')-tri-gold(I) at pressure

Pressure (GPa)	ambient	1.04	2.30	3.40	3.80	5.04	6.22	7.80
**Formula**	$C_9H_9N_6Au_3$	$C_9H_9N_6Au$	$C_9H_9N_6Au$	$C_9H_9N_6Au$	$C_9H_9N_6Au$	$C_9H_9N_6Au3$	$C_9H_9N_6Au_3$	$C_9H_9N_6Au_3$
**M_r**	792.12	792.12	792.12	792.12	792.12	792.12	792.12	792.12
**Crystal System**	Monoclinic	Monoclinic	Monoclinic	Monoclinic	Monoclinic	Monoclinic	Monoclinic	Monoclinic
**Space Group**	$P2_1/c$	$P2_1/c$	$P2_1/c$	$P2_1/c$	$P2_1/c$	$P2_1/c$	$P2_1/c$	$P2_1/c$
**a,b,c (Å)**	8.3340(12)<br>14.364(2)<br>11.0508(16)	8.1046(16)<br>13.919(3)<br>10.829(2)	7.9763(17)<br>13.543(3)<br>10.700(2)	7.9044(16)<br>13.295(3)<br>10.626(2)	7.8837(16)<br>13.217(3)<br>10.606(2)	7.8073(16)<br>13.026(3)<br>10.538(2)	7.7527(16)<br>12.866(3)<br>10.491(2)	7.6803(15)<br>12.717(3)<br>10.442(2)
**β (°)**	106.890(2)	107.27(3)	107.31(3)	107.26(3)	107.21(3)	107.26(3)	107.21(3)	107.23(3)
**V ($Å^3$)**	1265.9(3)	1166.4(4)	1103.4(4)	1066.4(4)	1055.7(4)	1023.4(4)	999.6(4)	974.2(3)
**Z/Z'**	4/1	4/1	4/1	4/1	4/1	4/1	4/1	4/1
**D_x (Mg m^{-3})**	4.156	4.511	4.768	4.934	4.984	5.141	5.263	5.401
**μ(mm^{-1})**	34.673	31.239	33.021	34.168	34.516	42.886	36.451	45.054
**F(000)**	1368	1368	1368	1368	1368	1368	1368	1368
**T_{min} /T_{max}**	0.3887/0.7468	0.7878/0.9686	0.7498/0.9739	0.7685/0.9408	0.8045/0.9679	0.7889/1.0000	0.8149/1.0000	0.7857/1.0000
**2θ range(°)**	2.78/34.68	2.31/ 29.43	2.84/29.57	2.89/29.57	2.91/29.47	2.41/29.55	2.43/29.58	2.45/29.60
**Rint**	0.0856	NA	NA	NA	NA	NA	NA	NA
**Completeness**	0.998	0.300	0.305	0.303	0.306	0.301	0.301	0.292
**No. Measured/ Independent Observed reflections**	13723/4012	1155/1155	1123/1123	1081/1081	1071/1071	1031/1031	1007/1007	955/955
**R[F]/wR[F2] (for I >2σ)**<br>**R[F]/wR[F2] (all data)**	0.0484/0.1347<br>0.0613/0.1491	0.0227/0.0469<br>0.0263/0.0481	0.0264/0.0704<br>0.0292/0.0722	0.0290/0.0981<br>0.0311/0.1007	0.0231/0.0626<br>0.0257/0.0641	0.0217/0.0551<br>0.0245/0.0563	0.0204/0.0430<br>0.0235/0.0440	0.0210/0.0412<br>0.0261/0.0424
**Restraints/Parameters**	0/164	30/88	30/88	30/88	30/88	30/88	30/88	30/88
**$\Delta\rho_{max}$,$\Delta\rho_{min}$ (e $Å^{-3}$)**	1.942/-2.170	0.658/-0.742	0.808/-0.851	0.985/-1.354	0.734/-0.868	0.657/-0.928	0.672/-0.844	0.896/-0.821
**REFCODE**		971088	971089	971090	971091	971092	971093	971094

Table 18 Crystallographic data of tris(μ_2-3,4,5-trimethylpyrazolato-N,N')-tri-gold(I) at pressure

**Pressure (GPa)**	**ambient**	**0.65**	**1.11**	**1.59**	**2.35**	**3.14**	**3.91**	**5.18**
**Formula**	$C_{18}H_{27}N_2Au_3$	$C_{18}H_{27}N_2Au_3$	$C_{18}H_{27}N_2Au_3$	$C_{18}H_{27}N_2Au_3$	$C_{18}H_{27}N_2Au_3$	$C_{18}H_{27}N_2Au_3$	$C_{18}H_{27}N_2Au_3$	$C_{18}H_{27}N_2Au_3$
**M_r**	918.36	918.36	918.36	918.36	918.36	918.36	918.36	918.36
**Crystal System**	Monoclinic	Monoclinic	Monoclinic	Monoclinic	Monoclinic	Monoclinic	Monoclinic	Monoclinic
**Space Group**	$P2_1/c$	$P2_1/c$	$P2_1/c$	$P2_1/c$	$P2_1/c$	$P2_1/c$	$P2_1/c$	$P2_1/c$
**a,b,c (Å)**	8.9753(2)<br>22.3828(6)<br>10.8274(3)	8.7372(18)<br>22.018(4)<br>10.483(2)	8.6507(17)<br>21.908(4)<br>10.356(2)	8.5716(17)<br>21.805(4)<br>10.237(2)	8.4772(17)<br>21.686(4)<br>10.062(2)	8.4092(17)<br>21.573(4)<br>9.913(2)	8.3405(17)<br>21.498(4)<br>9.765(2)	8.289(5)<br>21.438(5)<br>9.569(5)
**β (°)**	94.550(3)	93.08(3)	92.52(3)	92.00(3)	91.40(3)	90.60(3)	90.21(3)	89.582(5)
**V ($Å^3$)**	2168.29(10)	2013.8(7)	1960.7(7)	1912.1(7)	1849.2(6)	1798.2(6)	1750.9(6)	1700.4(14)
**Z/Z'**	4/1	4/1	4/1	4/1	4/1	4/1	4/1	4/1
**D_x (Mg m^{-3})**	2.813	3.029	3.111	3.190	3.299	3.390	3.484	3.587
**μ(mm^{-1})**	20.263	18.106	18.596	19.068	19.718	20.264	25.094	21.443
**F(000)**	1656	1656	1656	1656	1656	1656	1656	1656
**T_{min} /T_{max}**	0.70327/1.00000	0.5999/0.7660	0.8198/0.9730/	0.5343/0.7362	0.5343/0.7362	0.5546/0.8845	0.7859/0.9104	0.8001/0.9993
**2θ range(°)**	2.91/26.37	2.03/29.61	2.39/ 31.87	2.07/ 29.55	3.27/29.61	2.45/26.53	2.16/29.53	2.20/31.79
**Rint**	0.0474	NA	NA	NA	NA	NA	NA	NA
**Completeness**	0.999	0.264	0.298	0.307	0.299	0.324	0.290	0.280
**No. Measured/ Independent Observed reflections**	19262/4420	1783/ 1783	2118/ 2118	1956/ 1956	1861/1861	1447/1447	1695/1695	1729/1729
**R[F]/wR[F2] (for I >2σ)**<br>**R[F]/wR[F2] (all data)**	0.0320/0.0554<br>0.0561/0.0609	0.0327/ 0.0859<br>0.0434/ 0.0923	0.0340/ 0.0789<br>0.0495/ 0.0858	0.0365/ 0.0821<br>0.0514/ 0.0907	0.0472/0.1179<br>0.0569/0.1247	0.0440/0.1048<br>0.0612/0.1125	0.0298/0.0753<br>0.0342/0.0778	0.0426/0.0426<br>0.0551/0.1105
**Restraints/Parameters**	0/253	52/134	52/ 133	51/ 133	52/ 133	52/ 133	52/ 133	52/ 133
**$\Delta\rho_{max}$,$\Delta\rho_{min}$ (e $Å^{-3}$)**	1.359/-0.893	1.041/-0.820	1.090/-0.795	1.141/0.925	1.652/-2.589	1.175/-1.014	1.189/-1.369	2.980/-1.396
**REFCODE**		971081	971082	971083	971084	971085	971086	971087

Table 19. Crystallographic data of tris(μ_2-3-methyl-5-phenylpyrazolato-N,N')-tri-gold(I) at pressure

Pressure (GPa)	ambient	0.17
**Formula**	$C_{30}H_{27}N_6Au_3$	$C_{30}H_{27}N_6Au_3$
**M_r**	1062.48	1062.48
**Crystal System**	orthorhombic	orthorhombic
**Space Group**	*P n a 2₁*	*P n a 2₁*
**a,b,c (Å)**	7.4065(10)<br>16.001(2)<br>26.502(3)	7.2885(15)<br>15.843(3)<br>26.141(5)
**β (°)**	90	90
**V (Å^3)**	3140.9(7)	3018.5(11)
**Z/Z'**	4/1	4/1
**D_x (Mg m^{-3})**	2.247	2.338
**μ(mm^{-1})**	16.923	12.088
**F(000)**	1944	1944
**T_{min} /T_{max}**	0.5809/0.7469	0.8103/1.0000
**2θ range(°)**	2.80/29.64	2.90/28.59
**Rint**	0.0587	NA
**Completeness**	0.996	0.349
**No. Measured/ Independent Observed reflections**	43207/10119	3059/3059
**R[F]/wR[F2] (for I >2σ)**<br>**R[F]/wR[F2] (all data)**	0.0466/0.1177<br>0.0559/0.1224	0.0403/0.1090<br>0.0478/0.1144
**Restraints/Parameters**	1/355	69/175
**$\Delta\rho_{max}$,$\Delta\rho_{min}$ (e Å^{-3})**	1.075/-0.878	0.635/-0.728
**REFCODE**	971071	971072

Table 20. Crystallographic data of tris(μ_2-3,5-diphenylpyrazolato-N,N')-tri-gold(I) at pressure

Pressure (GPa)	ambient	0.52	0.97	1.24	1.31	1.76	2.05	2.31
**Formula**	$C_{45}H_{33}N_6Au_3$	$C_{45}H_{33}N_6Au_3$	$C_{45}H_{33}N_6Au_3$	$C_{45}H_{33}N_6Au_3$	$C_{45}H_{33}N_6Au_3$	$C_{45}H_{33}N_6Au_3$	$C_{45}H_{33}N_6Au_3$	$C_{45}H_{33}N_6Au_3$
**M_r**	1248.67	1248.67	1248.67	1248.67	1248.67	1248.67	1248.67	1248.67
**Crystal System**	Trigonal	Trigonal	Trigonal	Trigonal	Trigonal	Trigonal	Trigonal	Trigonal
**Space Group**	*R-3c*	*R-3c*	*R-3c*	*R-3c*	*R-3c*	*R-3c*	*R-3c*	*R-3c*
**a,b,c (Å)**	16.0783(3)<br>16.0783(3)<br>25.6914(7)	15.8344(7)<br>15.8344(7)<br>25.013(2)	15.7110(9)<br>15.7110(9)<br>24.560(3)	15.6768(7)<br>15.6768(7)<br>24.379(2)	15.6632(7)<br>15.6632(7)<br>24.327(2)	15.5942(7)<br>15.5942(7)<br>24.002(2)	15.5591(7)<br>15.5591(7)<br>23.825(2)	15.5377(7)<br>15.5377(7)<br>23.726(2)
**γ (°)**	120	120	120	120	120	120	120	120
**V (Å^3)**	5751.7(2)	5431.3(6)	5250.0(7)	5188.8(6)	5168.7(6)	5054.9(5)	4994.9(5)	4960.5(5)
**Z/Z'**	6/1/3	6/1/3	6/1/3	6/1/3	6/1/3	6/1/3	6/1/3	6/1/3
**D_x (Mg m^{-3})**	2.163	2.291	2.370	2.398	2.407	2.461	2.491	2.508
**μ(mm^{-1})**	11.491	10.086	10.434	10.557	10.598	10.837	10.967	11.043
**F(000)**	3492	3492	3492	3492	3492	3492	3492	3492
**T_{min} /T_{max}**	0.69800/1.0000	0.8275/1.0000	0.7835/1.000	0.8042/0.9931	0.7828/1.0000/	0.7671/1.0000	0.8055/1.0000	0.8118/1.0000
**2θ range(°)**	3.33/32.84	4.01/29.56	4.05/31.02	4.06/29.50	4.07/29.56	2.46/31.04	3.86/29.55	3.86/29.45
**Rint**	0.0367	0.0732	0.0743	0.0650	0.0728	0.0729	0.0674	0.0656
**Completeness**	0.992	0.673	0.626	0.578	0.671	0.642	0.642	0.585
**No. Measured/ Independent Observed reflections**	22942/2283	11870/1365	10948/1398	10931/1113	11327/1295	11360/1388	10670/1294	10485/1075
**R[F]/wR[F2] (for I >2σ)**<br>**R[F]/wR[F2] (all data)**	0.0264/0.0502<br>0.0395/0.0543	0.0338/0.0751<br>0.0458/0.0795	0.0399/0.1013<br>0.0514/0.1062	0.0300/0.0666<br>0.0367/0.0702	0.0295/0.0685<br>0.0393/0.0717	0.0323/0.0740<br>0.0461/0.0794	0.0320/0.0756<br>0.0402/0.0796	0.0286/0.0723<br>0.0374/0.0776
**Restraints/Parameters**	0/40	0/40	0/40	0/40	0/40	0/40	0/40	0/40
**$\Delta\rho_{max}$,$\Delta\rho_{min}$ (e Å^{-3})**	0.881/-0.924	0.888/-1.060	1.427/-1.042	0.786/-0.830	0.866/-0.841	0.844/-0.807	0.938/-0.861	1.028/-0.909
**REFCODE**	97110	971073	971074	971075	971076	971077	971078	971079

References

[1] C. F. Macrae, I. J. Bruno, J. A. Chisholm, P. R. Edgington, P. McCabe, E. Pidcock, L. Rodriguez-Monge, R. Taylor, J. van de Streek, P. A. Wood, *J. Appl. Crystallogr.* **2008**, *41*, 466-470.

[2] M. J. Cliffe, A. L. Goodwin, *J. Appl. Crystallogr.* **2012**, *45*, 1321-1329.

[3] R. Angel, EOSFIT version 5.2, Virginia Tech, Blackberg, 2002.

[4] P. K. Allan, B. Xiao, S. J. Teat, J. W. Knight, R. E. Morris, *J. Am. Chem. Soc.* **2010**, *132*, 3605-3611.

[5] P. A. Wood, J. J. McKinnon, S. Parsons, E. Pidcock, M. A. Spackman, *Crystengcomm* **2008**, *10*, 368-376.
